# The role of red and white light in optimizing growth and accumulation of plant specialized metabolites at two light intensities in medical cannabis (*Cannabis sativa* L.)

**DOI:** 10.3389/fpls.2024.1393803

**Published:** 2024-06-18

**Authors:** Mexximiliaan M. S. F. Holweg, Elias Kaiser, Iris F. Kappers, Ep Heuvelink, Leo F. M. Marcelis

**Affiliations:** ^1^ Horticulture and Product Physiology, Wageningen University, Wageningen, Netherlands; ^2^ Laboratory of Plant Physiology, Wageningen University, Wageningen, Netherlands

**Keywords:** medical cannabis, *Cannabis sativa* L., light spectrum, light intensity, photosynthetic photon flux density, plant specialized metabolites, morphology, photosynthesis

## Abstract

The cultivation of medical cannabis (*Cannabis sativa* L.) is expanding in controlled environments, driven by evolving governmental regulations for healthcare supply. Increasing inflorescence weight and plant specialized metabolite (PSM) concentrations is critical, alongside maintaining product consistency. Medical cannabis is grown under different spectra and photosynthetic photon flux densities (PPFD), the interaction between spectrum and PPFD on inflorescence weight and PSM attracts attention by both industrialists and scientists. Plants were grown in climate-controlled rooms without solar light, where four spectra were applied: two low-white spectra (7B-20G-73R/Narrow and 6B-19G-75R/2Peaks), and two high-white (15B-42G-43R/Narrow and 17B-40G-43R/Broad) spectra. The low-white spectra differed in red wavelength peaks (100% 660 nm, versus 50:50% of 640:660 nm), the high-white spectra differed in spectrum broadness. All four spectra were applied at 600 and 1200 μmol m^-2^ s^-1^. Irrespective of PPFD, white light with a dual red peak of 640 and 660 nm (6B-19G-75R/2Peaks) increased inflorescence weight, compared to white light with a single red peak of 660 nm (7B-20G-73R/Narrow) (tested at *P* = 0.1); this was associated with higher total plant dry matter production and a more open plant architecture, which likely enhanced light capture. At high PPFD, increasing white fraction and spectrum broadness (17B-40G-43R/Broad) produced similar inflorescence weights compared to white light with a dual red peak of 640 and 660 nm (6B-19G-75R/2Peaks). This was caused by an increase of both plant dry matter production and dry matter partitioning to the inflorescences. No spectrum or PPFD effects on cannabinoid concentrations were observed, although at high PPFD white light with a dual red peak of 640 and 660 nm (6B-19G-75R/2Peaks) increased terpenoid concentrations compared to the other spectra. At low PPFD, the combination of white light with 640 and 660 nm increased photosynthetic efficiency compared with white light with a single red peak of 660nm, indicating potential benefits in light use efficiency and promoting plant dry matter production. These results indicate that the interaction between spectrum and PPFD influences plant dry matter production. Dividing the light energy in the red waveband over both 640 and 660 nm equally shows potential in enhancing photosynthesis and plant dry matter production.

## Introduction

Medical cannabis (*Cannabis sativa* L.) has gained prominence in both the horticultural and pharmaceutical industries due to its pharmacologically active compounds, notably cannabinoids and terpenoids (Karst et al., 2003; Andre et al., 2016; McPartland, 2018). These plant specialized metabolites (PSM) are primarily localized in the glandular trichomes on female inflorescences (Livingston et al., 2020). Medical cannabis is predominantly prescription-based and is endorsed for a variety of medical conditions including chronic neuropathic pain, nausea, vomiting, spasticity associated with multiple sclerosis, anorexia in cancer or HIV/AIDS patients, and symptoms of Tourette’s syndrome (De Hoop et al., 2018). While terpenoids possess medicinal attributes, they are primarily noted for their contributions to the aroma and flavor profiles of medical cannabis (Booth and Bohlmann, 2019; Sommano et al., 2020). The cannabinoids serve as the basis for classifying medical cannabis varieties into five distinct chemotypes, determined by the ratio of their dominant cannabinoids, Delta-9-Tetrahydrocannabinol (THC) to Cannabidiol (CBD) (Aizpurua-Olaizola et al., 2016).

Controlled conditions are essential for maintaining consistent production in medical cannabis, in terms of inflorescence yield and PSM concentrations. Considering that unprocessed inflorescences are directly administered to patients, it is of critical importance to achieve uniform PSM concentrations and consistent pharmacological effects (Hazekamp et al., 2006; Kowal et al., 2016). Cultivation in controlled environment conditions without solar light demands substantial energy inputs, particularly for lighting (Zobayed et al., 2005; Mehboob et al., 2020). The expansion of medical cannabis industries globally underscores the necessity for energy-efficient lighting systems (Hall et al., 2019). A transition is occurring from conventional lighting systems, such as fluorescent and high-intensity discharge lamps, to more energy-efficient light-emitting diode (LED) technology (Mitchell et al., 2015; Pattison et al., 2018; Kusuma et al., 2020). LED technology offers benefits such as enhanced energy efficacy, lifespan, and spectrum customization, while maintaining high photosynthetic photon flux densities (PPFD) with reduced heat emission, thus enabling effective manipulation of light to influence plant dry matter production and development (Morrow, 2008; Burgie et al., 2014; Galvão and Fankhauser, 2015; Ouzounis et al., 2015; Krahmer et al., 2018).

Light spectrum affects plant dry matter production and metabolic processes, through photoreceptors including phytochromes, cryptochromes, phototropins, and UVR8 (Folta & Carvalho, 2015; Ouzounis et al., 2015; Pocock, 2015; Thoma et al., 2020), as well as through its effects on net photosynthesis rate (*A*; e.g. McCree, 1971; Hogewoning et al., 2012). Most studies focused on evaluating the individual effects of either spectrum or PPFD on inflorescence weight and PSM concentrations (Magagnini et al., 2018; Eaves et al., 2020; Llewellyn et al., 2021; Rodriguez-Morrison et al., 2021; Danziger and Bernstein, 2021a; Islam et al., 2022). However, there is a growing recognition, observed in other plant species, that spectrum and PPFD interactively influence plant dry matter production and PSM concentrations (Cope & Bugbee, 2013; Ouzounis et al., 2015; Snowden et al., 2016; Eichhorn Bilodeau et al., 2019). LED lighting systems were primarily characterized by peak wavelengths around 660 nm, as these were the first LEDs with adequate output for plant dry matter production (Bula et al., 1991; Morrow, 2008). The peak wavelength of 660 nm closely corresponds with the maximum absorption wavelength of Chlorophyll *a* (Chl *a*; 663 nm), and as Chl *a* is the predominant pigment in the reaction centers of both photosystem II (PSII) and photosystem I (PSI), this alignment has justified the mass production and adoption of 660 nm LEDs in horticulture since 1991 (Tamulaitis et al., 2005; Caffarri et al., 2011; Zhao et al., 2017; Pan et al., 2018; Bula et al., 1991). Exploiting the local absorption peak of Chlorophyll *b* (Chl *b*; 642 nm) could enhance light use efficiency and light absorption further, as Chl *b* is essential in preventing photoinhibition and improving energy transfer between the light-harvesting- and photosystem core complexes (Voitsekhovskaja and Tyutereva, 2015). This is particularly relevant as the highest luminous efficiency in the red region occurs at 640 nm, and aligns with the region of the highest photosynthetic quantum yield, which spans from 600 to 660 nm (McCree, 1971; Inada, 1976; Evans, 1987; Tamulaitis et al., 2005; Hogewoning et al., 2012). Wollaeger and Runkle (2013) investigated the effects of combinations of two wavelengths within the red waveband (634 and 664 nm), with the addition of 10% blue (446 nm) and 10% green (516 nm), on various crops grown at PPFD of 125 and 250 μmol m^-2^ s^-1^. Their findings indicated limited morphological differences under these conditions. However, it is important to note that medical cannabis, which is often cultivated at significantly higher PPFD, may respond differently.

The spectrum of LED, particularly the red-to-blue ratio, varies in horticultural applications and is crucial for plant dry matter production and development (Kim et al., 2004; Piovene et al., 2015). Red photons are generally more efficient in driving *A*, as they are less strongly absorbed by non-photosynthetic pigments compared to blue and green photons (Emerson and Lewis, 1943; McCree, 1971; Inada, 1976; Farquhar et al., 1980; Evans, 1987). Also, exposure to high PPFD can lead to overexcitation of the photosystems, potentially causing photoinhibition (Miao et al., 2016; Oguchi et al., 2021). Furthermore, overexcitation of pigments, notably under low-white spectra, has been suggested to be associated with the appearance of bleached inflorescences—a loss of pigmentation in the apical inflorescence that adversely impacts marketability (Hawley, 2023). Incorporating a higher white fraction, resulting in a more balanced red-to-blue ratio and increased green fraction, may reduce the risk of photoinhibition within the palisade layer due to increased light penetration within the leaf, and thus foster higher quantum yields at higher PPFD (Terashima et al., 2009; Oguchi et al., 2011, 2021). Such a strategy facilitates a more balanced distribution of light absorption across photosynthetic and non-photosynthetic pigments, thereby decreasing the risk of photoinhibition (Tracewell et al., 2001; Terashima et al., 2009; Hogewoning et al., 2012). Furthermore, LED fixtures exhibit variability in their spectra, ranging from narrow to broad bandwidths. Broadband spectra, offering a more even distribution of light across a wider range of wavelengths, may be more effective in providing balanced light exposure for *A* and plant dry matter production (Hogewoning et al., 2012). Variations in plant responses due to spectra, coupled with their interplay with PPFD, highlight the necessity of selecting an appropriate lighting system tailored to the specific requirements of medical cannabis.

The influence of spectrum on PSM concentrations in medical cannabis has been explored in various studies (Eichhorn Bilodeau et al., 2019; Westmoreland et al., 2021; Islam et al., 2022). While exposure to blue light was correlated with increased cannabinoid concentrations (Hawley et al., 2018; Namdar et al., 2019; Danziger and Bernstein, 2021a), opposite effects were found as well (Wei et al., 2021; Westmoreland et al., 2021). These discrepancies may arise from the use of varying PPFD across studies. In other plant species, both red and blue light have been shown to affect terpenoid concentrations, and this might provide insights for terpenoid production in medical cannabis (Kessler and Kalske, 2018; Ghaffari et al., 2019). Further, while instantaneous responses of *A* in medical cannabis to PPFD, temperature, and [CO_2_] are well-documented (Chandra et al., 2008, 2011a, 2011b, 2015), the effects of photosynthetic acclimation to different spectra remain unexplored (Liu and Van Iersel, 2021).

Despite a broad array of spectra and PPFD applied by horticulturists, a significant knowledge gap exists on the effects of these factors in medical cannabis. Previous studies have explored the impact of a single spectrum, leaving room for further investigation into the effects of spectra (Eaves et al., 2020; Llewellyn et al., 2021; Rodriguez-Morrison et al., 2021). Some efforts to clarify this relationship encountered complexities, notably the difficulty in maintaining consistent PPFD across different spectral treatments (Magagnini et al., 2018; Danziger and Bernstein, 2021a; Morello et al., 2022). This study aims to investigate the effects of different red wavelengths (640 and 660 nm), white fraction, and spectrum broadness on plant dry matter production and partitioning, and specialized metabolite accumulation in medical cannabis. It focuses on comprehensively analyzing plant morphology and photosynthetic responses at both low (600 μmol m^-2^ s^-1^) and high (1200 μmol m^-2^ s^-1^) PPFD, to clarify the underlying mechanisms of spectrum-PPFD interactions.

## Material and methods

### Plant material and propagation growth conditions

**Figure 1:**
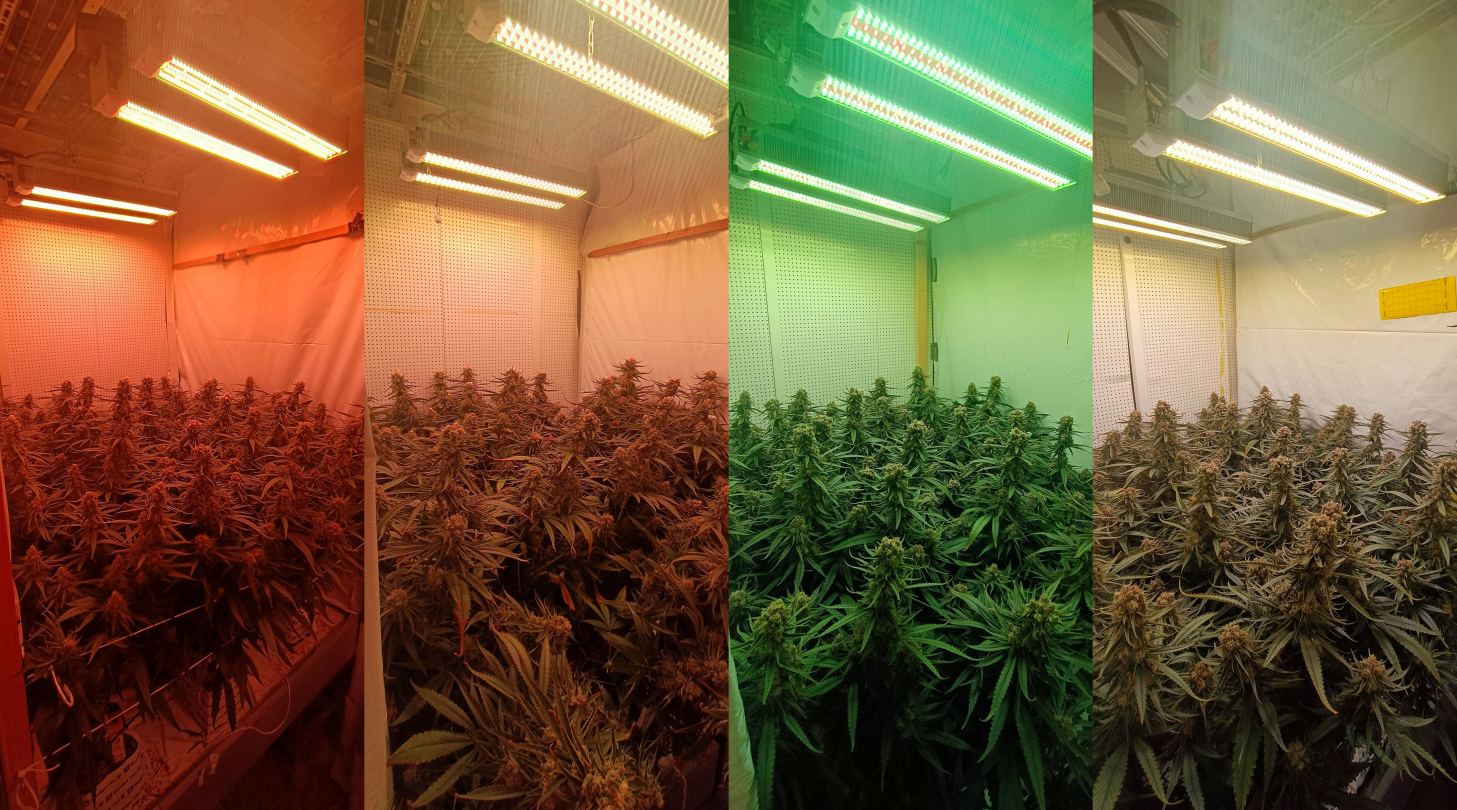
Photographs of Cannabis sativa under the treatment spectra, 42 days after start of the short-day phase, 56 days after transplanting. Spectra are displayed from left to right as follows: 6B-19G-75R/2Peaks, 7B-20G-73R/Narrow, 15B-42G-43R/Narrow, and 17B-40G-43R/Broad.

C*annabis sativa* plants (var. King Harmony (Chemotype II, 1:1.5 THC : CBD); Perfect Plants, Honselersdijk, the Netherlands) were cultivated in two sequential growth cycles in climate-controlled chambers (**Figure 1**). These chambers were each divided into eight sections utilizing white plastic sheets. Genetically identical mother plants, derived from tissue culture and younger than four months, provided 228 unrooted apical cuttings, measuring 10 cm in length and possessing one fully expanded leaf with excised axillary nodes. These cuttings were propagated according to a standard propagation protocol (Text S1).

### Growth conditions during vegetative and generative phase

At day 21 of the propagation phase, a uniform selection of 128 plants was transplanted into 15 x 15 x 15 cm stone wool blocks (Hugo Blocks; Grodan) and grown at a planting density of 16 plants m^-2^ for 14 days under long days (18 h photoperiod); plants achieved a height of 30 cm. Subsequently, plants were grown for 56 days at a planting density of 9 plants m^-2^ during the short-day phase (12 h photoperiod), to induce flower development.

Twenty-four hours prior to transplanting, stone wool plugs and blocks were pre-soaked in a nutrient solution (**Supplementary Table S1**) with electrical conductivity (EC) of 1.5 and 2.2 dS m^-1^, respectively. The pH of the nutrient solution was ~5.8. Stone wool plugs were irrigated on day 14 of the propagation phase by ebb- and flow. A drip irrigation system administered the nutrient solution six and four times daily for the long-day and short-day phase, respectively at a rate of 60 mL min^-1^ and a duration between two and four minutes, per stone wool block, depending on the irrigation demand for healthy plant growth. EC values of these nutrient solutions were 2.2 and 2.5 dS m^-1^ for the long-day and short-day phase, respectively (**Supplementary Table S1**).

**Figure 2:**
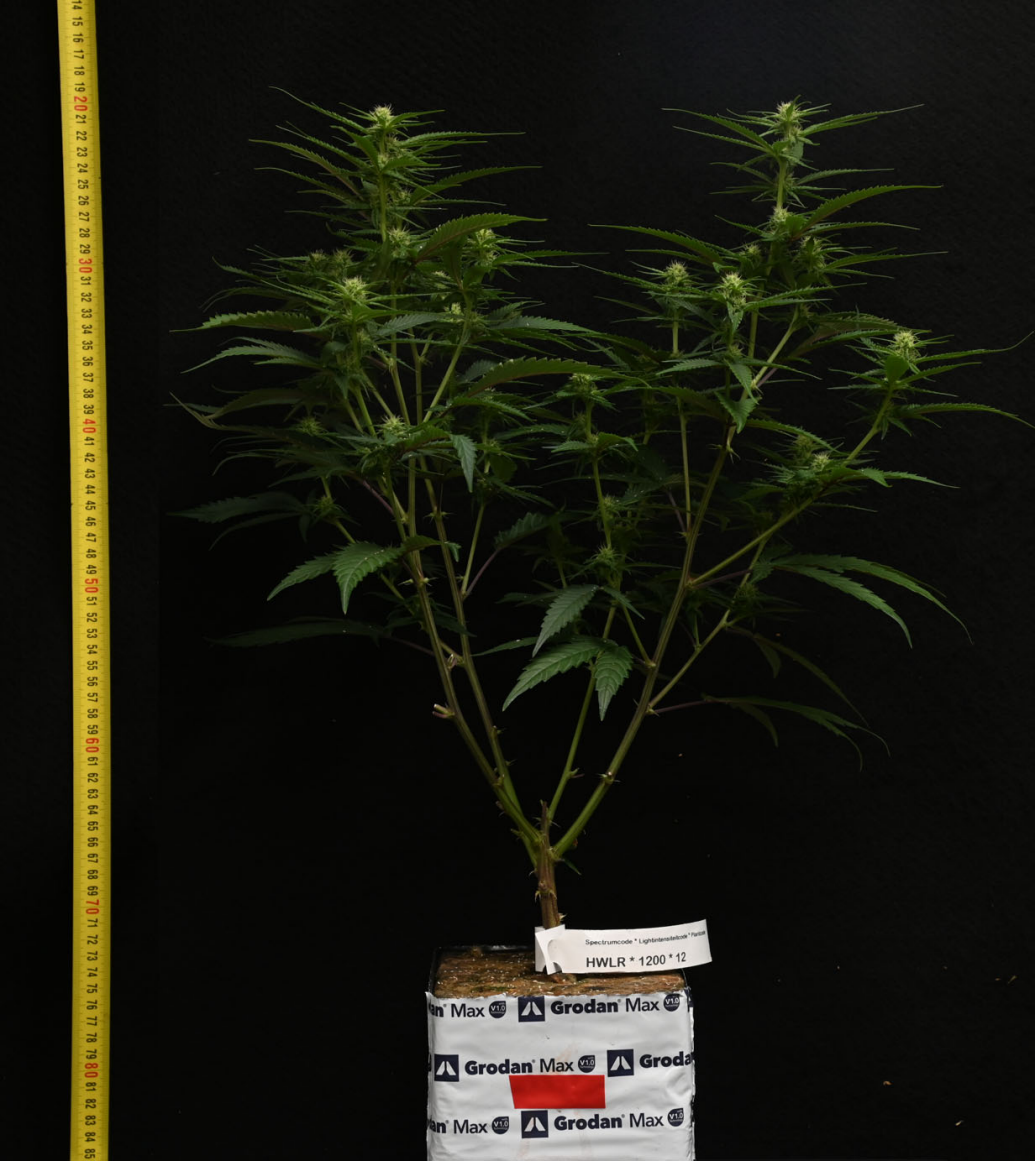
Representative image of Cannabis sativa after pruning 10 days after start of the short-day phase, 24 days after transplanting.

At day 7 of the long-day phase, four secondary branches per plant were retained, to improve crop uniformity and reduce apical dominance, by removing the apical meristem at the seventh node and removing the two lowest secondary branches. At day 10 of the short-day phase, plants were pruned to promote airflow and reduce a high relative humidity in the canopy’s microclimate by removing the bottom 20 cm of leaves and tertiary branches (**Figure 2**); pruned plant material was collected for inclusion in total plant dry matter production. RH was 75% and decreased to 70% on day 7 of the long-day phase to promote transpiration. For the short-day phase, relative humidity was set to 65% and subsequently decreased by 5% weekly until it reached 55% to promote transpiration and thus water uptake, and to prevent infections and infestations such as Botrytis (*Botrytis cinerea*) and powdery mildew (*Golovinomyces ambrosiae* and *Podosphaera macularis*). Air temperature was set to 28/24°C, 27/22°C, 26/22°C, and 25/22°C during the long-day phase, and on days 0-28, 29-42, and day 49-56 of the short-day phase, respectively. This temperature regime aimed to facilitate generative growth. [CO_2_] was set to 800/400 and 1000/400 ppm (day/night) during the long-day and short-day phase, respectively.

### Light treatments

**Figure 3:**
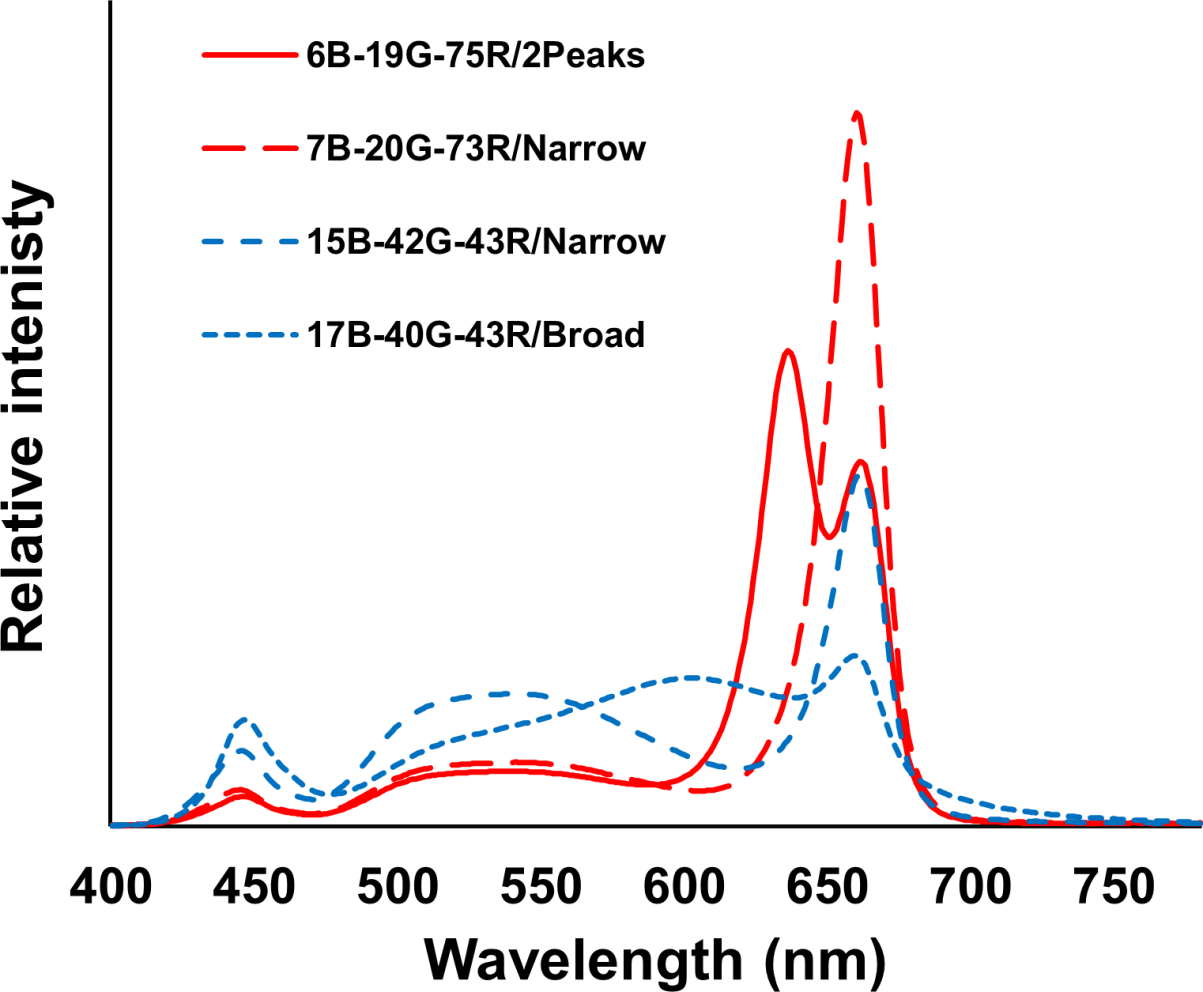
Spectral distribution of the four studied spectra with their quantitative parameters shown in **Table 1**.

The PPFD at canopy height was 600 and 1200 µmol m^-2^ s^-1^ (26 and 52 mol m^-2^ d^-1^, respectively), provided by LEDs (ams OSRAM, Munich, Germany) mounted in VYPR fixtures (Fluence, Texas, Austin, USA). Four spectra were applied at both PPFD: two low-white (7B-20G-73R/Narrow and 6B-19G-75R/2Peaks) and two high-white (15B-42G-43R/Narrow and 17B-40G-43R/Broad) spectra (**Figure 3**). The blue-green-red ratios of the two low-white spectra were approximately equivalent, as well as the ratios of the two high-white spectra (**Table 1**). The low-white spectra either contained a single peak wavelength at 660 nm (7B-20G-73R/Narrow) or dual peak wavelengths at 640 and 660 nm (6B-19G-75R/2Peaks). The high-white spectra differed in broadness of the white spectrum: narrowband spectrum (42G-43R/Narrow), featuring peak wavelengths at 450 nm and 660 nm, and broadband spectrum (17B-40G-43R/Broad), which displayed a more uniform light distribution across a wide range of wavelengths, approximately spanning 400-750 nm.

**Table 1:** Spectral distribution and Photosynthetic Photon Efficacy (PPE) of the four spectra studied: 6B-19G-75R/2Peaks, 7B-20G-73R/Narrow, 15B-42G-43R/Narrow, and 17B-40G-43R/Broad and ratios of red to blue (R:B), red to green (R:G), blue to green (B:G), and red to far-red (R:FR).

Spectrum	% of total PPFD (400-700 nm)			% of PFD		Ratio				PPE (µmol/J)
				(380-780 nm)						
	Blue	Green	Red	Ultraviolet	Far Red	R:B	R:G	B:G	R:FR	
**6B-19G-75R/2Peaks**	6	19	75	0.1	1	12	0.2	0.3	69	3.31
**7B-20G-73R/Narrow**	7	20	73	0.1	1	10.2	0.3	0.4	56.2	3.44
**15B-42G-43R/Narrow**	15	42	43	0.1	1	2.8	1	0.4	31.8	2.91
**17B-40G-43R/Broad**	17	40	43	0.1	3	2.6	0.9	0.4	12.7	3.06

During the long-day phase, the PPFD was initially set at 400 µmol m^-2^ s^-1^ and gradually increased to 600 µmol m^-2^ s^-1^ by day 12. In the short-day phase, the PPFD was further increased to 1200 µmol m^-2^ s^-1^ on day 7 for half of the plots. Weekly quantum sensor measurements (MQ-610, Apogee Instruments Inc., Logan, CA, USA) were conducted across nine points per plot to ensure uniform PPFD at canopy height.

### Destructive measurements

Per treatment, seven plants per plot were destructively measured at the transition from the long-day to the short-day phase (14 days after transplanting), and nine plants were measured at the end of the experiment (70 days after transplanting). Dry weights of inflorescences, leaves that had been trimmed from the inflorescences, regular leaves, and stems were quantified. Inflorescence weights were determined after trimming inflorescence leaves with an industrial trimmer (MT Tumbler 200; Master Products, Girona, Spain). Leaf area of regular leaves was determined using a LI-3100C area meter (LI-COR Inc., Lincoln, Nebraska, USA). Dry weight was determined using a ventilated oven (24h at 70°C, followed by 48h at 105°C). Inflorescence weight at 10% moisture content was calculated from the oven dry weight of the inflorescence by multiplication with 1.10. Inflorescence length and width were measured on each of the four branches per plant to calculate inflorescence volume (assuming a cylinder shape) by $inflorescence\;volume=inflorescence\;length*\;\pi *{\left(\frac{inflorescence\;width}{2}\right)}^{2}$ . The inflorescence is identified as the complete inflorescence structure on a single branch (Spitzer-Rimon et al., 2019). Inflorescence density was calculated by dividing inflorescence dry weight by inflorescence volume. Light use efficiency (LUE) was determined by dividing inflorescence or total plant dry weight by the cumulative incident photosynthetically active radiation (PAR) at canopy height, across both the long-day and short-day phases (total light integral; TLI).

### Leaf light absorptance, transmittance, and reflectance

Leaf light absorptance was measured in accordance to (Taylor et al., 2019), which involved the use of a dark enclosure equipped with two integrating spheres to determine leaf light transmission and reflection. Per treatment, leaf samples were collected from six randomly selected plants, with one leaf per plant, to quantify leaf light absorptance. Selected leaves were fully expanded, containing five or more leaflets, and positioned within 20 cm from the apex, ensuring full exposure to the light. The calculation of absorbed PAR (PAR_abs_) involved multiplying incident PAR by leaf light absorptance.

### Leaf photosynthesis measurements

Leaf photosynthesis was measured using a LI-6800 photosynthesis system (LI-COR) on six randomly selected plants per treatment (6 replicate plants per plot). Gas-exchange measurements were conducted on leaves that were selected on similar criteria as for leaf light absorptance. Data were collected during the fourth and seventh week of the short-day phase. Measurements of leaf photosynthesis light-response curves and operational photosynthesis were conducted within a seven-hour window per measurement day, starting one hour after the lights turned on. Measurements were alternated between treatments to reduce possible time-of-day effects. The infrared gas analyzers were matched between measurements on different plants. Conditions within the fluorometer cuvette were set to 27°C, 60% RH, a fan speed of 10000 rpm, a flow rate of 400 µmol s^-1^, 2000 ppm [CO_2_], and spectrum of 20B:80R. Following a 15-minute light acclimation period to 3000 µmol m^-2^ s^-1^, *A* was stabilized and recorded for 120-180 s, depending on the stabilization of *A*. Sequential PPFD were set to: 3000, 2500, 2000, 1500, 1000, 800, 600, 400, 200, 100, and 0 µmol m^-2^ s^-1^. A non-rectangular hyperbola was fitted to the light response curve data (Thornley, 1977), and the parameters maximum net assimilation rate at saturating light (*A*_max_), quantum yield (α_LRC_), light compensation point (LCP), and dark respiration rate (R_d_) were obtained.

Measurements of operational photosynthesis (*A*_op_) were obtained using a transparent leaf cuvette at the incident PAR at canopy height. Environmental conditions within the leaf cuvette were set equal to the climate room environment. Quantum yield of photosynthesis (α_op_) was calculated as ${\text{α}}_{op}=\frac{\left(A_{op}+\;R_{d}\right)}{PAR_{abs}s}$ , where R_d_ is the average respiration rate per plot, estimated from the light-response curve.

### Gas chromatography-mass spectrometry analysis

Cannabinoid and terpenoid concentrations were quantified in inflorescences located at the apical inflorescence above the canopy, within 5 cm from the apical inflorescence. Per treatment, three pooled samples were collected, each three samples at four different times, 0, 5, 10, and 15 days before harvest (DBH). Each pooled sample consisted of three inflorescence clusters, each harvested from a randomly selected plant of a given treatment, totaling approximately 1 g per pooled sample. Bleached inflorescences, which were exclusively found at the tip of the apical inflorescences in the 6B-19G-75R/2Peaks treatment at 1200 µmol m^-2^ s^-1^, were individually harvested and analyzed, with each sample weighing approximately 0.4 g. Inflorescence samples were stored at -80°C until further processing. Per sample, 0.2 ± 0.01 g were measured into a glass tube, into which 2 mL of *n*-Hexane with 1 mg L^-1^ squalene (Thermo Fisher Scientific, Waltham, Massachusetts, USA) as an internal standard was added. Extraction of PSM was performed for 10 minutes, using an ultrasonic bath without elevated temperatures (Branson 2800; Branson Ultrasonics Corporation, Danbury, CT, USA). The resulting extract was then passed through a filtration column, containing of a Pasteur’s pipet filled with glass-wool and anhydrous sodium sulphate (Biosolve B.V., Valkenswaard, the Netherlands), and collected in a 2 ml glass vial for Gas Chromatography-Mass Spectrometry (GC-MS) analysis.

The PSM analysis was conducted using an Agilent Gas Chromatography (GC) Model 7890 (Agilent Technologies, Inc., Santa Clara, CA, USA) system fitted with a 30 x 0.25 mm i.d., 0.25-µm film thickness Zebron 5MS Column (Phenomenex Inc., Torrance, CA, USA), and a Model 5972A Mass Selective (MS) Detector (Hewlett-Packard, Palo Alto, CA, USA). The GC was programmed at an initial temperature of 60°C for two minutes, increased by 5°C min^−1^ until reaching 250°C, accelerated at 10°C min^−1^ to 280°C, and kept at this temperature for 5 min. The temperatures of the injection port, interface, and MS source were set to 250°C, 290°C, and 180°C, respectively. Helium inlet pressure was electronically controlled to sustain a constant column flow rate of 1.0 ml min^−1^. Ionization was conducted at a potential of 70 eV, and mass scanning ranged from 45 to 400 amu with a scan rate of 5 scans min^-1^. Samples were diluted 5-fold (i.e. 0.2 ml extract combined with 0.8 ml n-hexane) and one µL of each sample was injected and analyzed in split less mode.

Identification of terpenoid and cannabinoid compounds was based on their respective GC-MS retention times, and spectral comparisons against the NIST11 Mass Spectral Library (National Institute of Standards and Technology, Gaithersburg, MD, USA), the Adams essential oil library (Sparkman, 2005) and a comprehensive *in-house* spectral library generated with authentic standards. For semi-quantification of compounds, areas under the curve (AUC) were computed relative to the AUC of the internal standard (Squalene) and normalized for dilution and sample weight. For each treatment, data are presented as the mean ± SEM derived from three replicates. Concentrations of THC and CBD were determined using calibration curves from authentic standards, while additional cannabinoids and terpenoids were quantified in units relative to the internal standard. Initial quantifications were based on fresh weight, which were then normalized to a 10% moisture content, which reflects the market-standard weight for saleable inflorescences, by accounting for dry matter in the inflorescences. The THC and CBD yields were determined by multiplying their respective cannabinoid concentrations by the dry weight of the inflorescence.

### Statistical analysis

The experiment was set up and analyzed as a split-plot design in two blocks (repetition over time) with PPFD (600 and 1200 µmol m^-2^ s^-1^) as main factor and spectrum (6B-19G-75R/2Peaks, 7B-20G-73R/Narrow, 15B-42G-43R/Narrow, and 17B-40G-43R/Broad) as subfactor. Each plot consisted of 16 plants, of which seven were harvested at an intermediate harvest, and nine at the final harvest. Individual plant responses were averaged per plot and an average was used as a statistical replicate. Due to the limited number of blocks, homogeneity of variances had to be assumed and statistical significance was assessed at *P* = 0.1, which is consistent with standard practices in such conditions (Ott and Longnecker, 2015; Kaiser et al., 2019). No outliers were identified per plot, using Z-score criteria, with thresholds set at -3 and +3 standard deviations. A Shapiro-Wilk test ascertained that the assumptions of normality were met. Analysis of variance (ANOVA) was conducted to evaluate main and interaction effects of spectrum and PPFD on plant morphological traits, physiological traits, and PSM. Fisher’s unprotected LSD test was used for means separation. The variance in treatment effects on morphological parameters between the two repetitions could be attributed to an infection of Hop Latent Viroid in the first repetition. This infection, confirmed by Naktuinbouw in Roelofarendsveen, the Netherlands, likely diminished the observed treatment effects. Plants infected with Hop Latent Viroid exhibit symptoms including stunted growth and reduced inflorescence yield (Adkar-Purushothama et al., 2023). The variations observed may also be partly attributed to an earlier harvest by two weeks in the first repetition, necessitated by a malfunction of the irrigation system. To ensure comparability between the two experimental repetitions, PSM concentrations are presented only for 15 DBH for both repetitions. For a similar reason, photosynthesis data are primarily discussed for the fourth week of the short-day phase, as for the seventh week of the short-day phase only data from one repetition was available. Instances where data from only one repetition are presented are explicitly indicated. Statistical analysis was conducted by using SPSS (Version 26.0; IBM Corp., Armonk, NY, USA).

## Results

### Plant dry matter production and development

**Figure 4:**
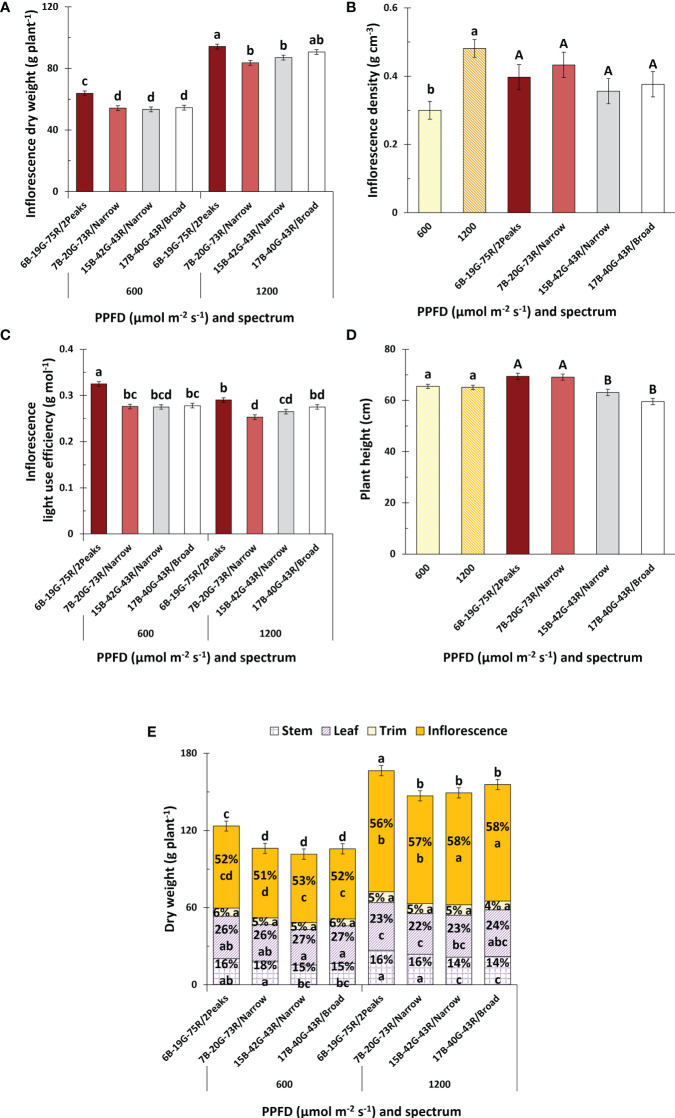
Effects of spectrum and PPFD on yield and light use efficiency of *Cannabis sativa*. **(A)** inflorescence dry weight; **(B)** inflorescence density; **(C)** inflorescence light use efficiency; **(D)** plant height; **(E)** and plant dry weight and partitioning. Bars indicate means of two blocks (n = 2) each consisting of 9 replicate plants. Main effects are shown when no interaction is found. Error bars represent standard error of means (SEM). Different letters (within lowercase and uppercase) indicate significant differences between treatments (Fisher’s unprotected LSD test, *P* = 0.10).

**Figure 5:**
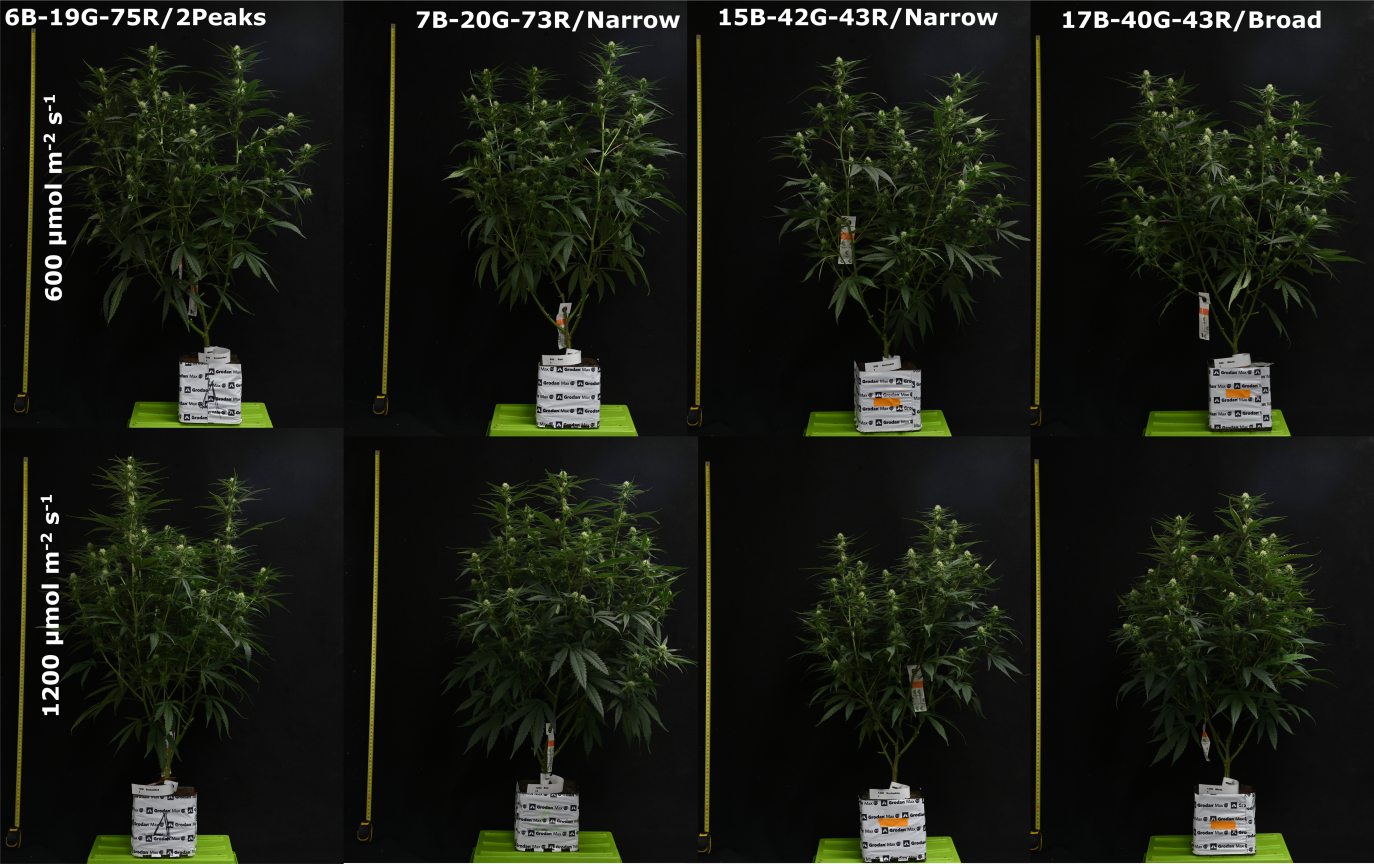
Representative images of Cannabis sativa 20 days after start of the short-day phase, 34 days after transplanting.

White light with a dual red peak of 640 and 660 nm (6B-19G-75R/2Peaks) increased inflorescence weight compared to white light with a single red peak at 660 nm (7B-20G-73R/Narrow), irrespective of PPFD (**Figure 4A**). This increase in inflorescence weight was related to an increase in total plant weight, while dry matter partitioning to inflorescences remained unaffected (**Figure 4E**). Neither increasing the white fraction (15B-42G-43R/Narrow compared to 7B-20G-73R/Narrow) nor increasing spectrum broadness (17B-40G-43R/Broad compared to 15B-42G-43R/Narrow) affected inflorescence weight at either PPFD. Dry matter partitioning to the inflorescences increased when the white fraction increased, irrespective of PPFD (**Figure 4E**). There was no effect of red wavelength or spectrum broadness on dry matter partitioning to the inflorescences. Dry matter partitioning to the trim and leaves was not affected by spectrum or PPFD. Increasing the white fraction reduced dry matter partitioning towards the stem, irrespective of PPFD. This coincided with a decrease in plant height (**Figure 4D**), resulting in a more compact plant architecture (**Figure 5**). Leaf area was not affected by spectrum, and decreased with increasing PPFD (**Supplementary Figure S2C**). Specific leaf area decreased with increasing spectrum broadness at higher PPFD, and generally decreased with increasing PPFD (**Supplementary Figure S2D**). There were no effects of spectrum on plant height, specific leaf area, leaf area, and total plant weight and biomass partitioning at intermediate harvest (**Supplementary Figure S1**). Inflorescence and plant LUE increased under white light with a dual red peak of 640 and 660 nm compared to white light with a single red peak at 660 nm, and decreased with increasing PPFD for this treatment (**Figure 4C** and **Supplementary Figure S2B**). Neither increasing the white fraction nor spectrum broadness affected inflorescence and plant LUE, and interestingly, both LUE were also unaffected by PPFD. Furthermore, inflorescence density increased with increasing PPFD, but was unaffected by spectrum (**Figure 4B**).

### Plant specialized metabolites

**Figure 6:**
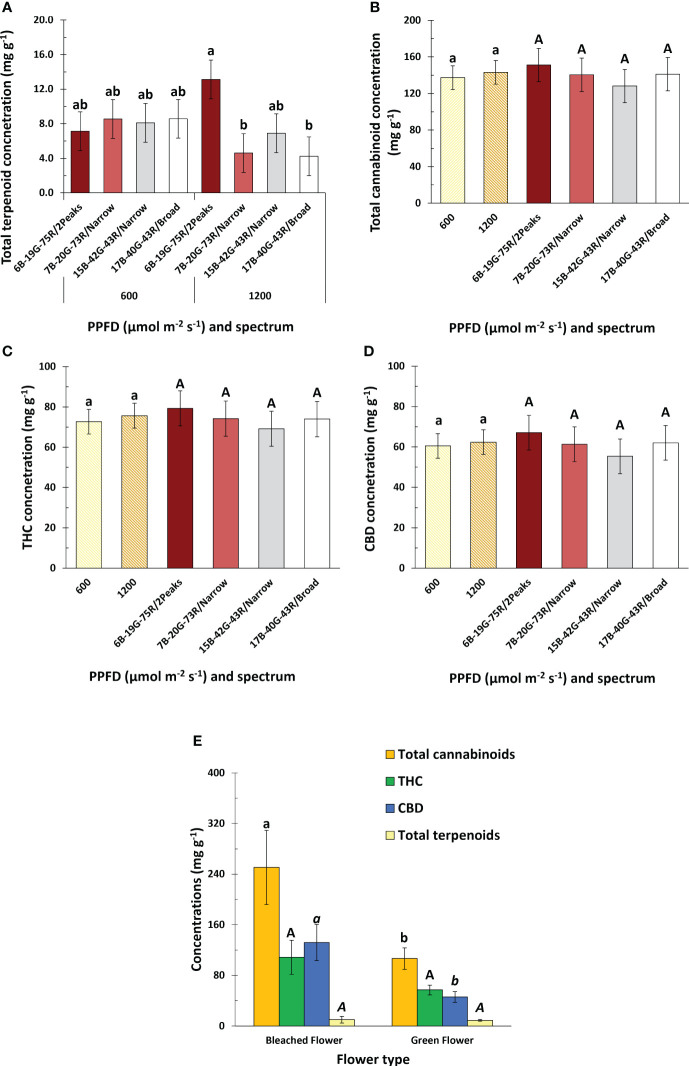
Effects of spectrum and PPFD on specialized metabolite concentration of *Cannabis sativa*. **(A)** total terpenoid concentration; **(B)** total cannabinoid concentration; **(C)** THC concentration; **(D)** CBD concentration. **(E)** effect of inflorescence type on total cannabinoid, THC, CBD, and total terpenoid concentration. Bars indicate means of two blocks (n = 2) each consisting of 9 replicate plants, with the exception of panel **(E)** which only consisted of one block. Main effects are shown when no interaction is found. Error bars represent standard error of means (SEM). Different letters (within lowercase and uppercase) indicate significant differences between treatments (Fisher’s unprotected LSD test, *P* = 0.10).

Spectrum or PPFD did not affect total cannabinoid concentration, nor that of any specific cannabinoid (**Figure 6B** and **Supplementary Table S2**). White light with a dual red peak of 640 and 660 nm compared to white light with a single red peak at 660 nm increased total terpenoid concentrations at high PPFD (**Figure 6A**). Neither increasing the white fraction nor spectrum broadness, irrespective of PPFD, affected total terpenoid concentrations. Total terpenoid concentration was manifested predominantely by β-Myrcene, α-Pinene, β-Pinene, Limonene, and Germacrene D (**Supplementary Table S2**), and was highest 5 days before harvest (DBH) (**Supplementary Figure S3B**). Bleached inflorescences were exclusively found at the tip of apical inflorescences in white light with a dual red peak of 640 and 660 nm at 1200 µmol m^-2^ s^-1^, and not in the other treatments. Bleached inflorescences exhibited increased total cannabinoid concentrations compared to green inflorescences, primarily attributed to CBD as THC was not affected (**Figure 6E**). The type of inflorescence did not influence total terpenoid concentrations.

### Photosynthesis

**Figure 7:**
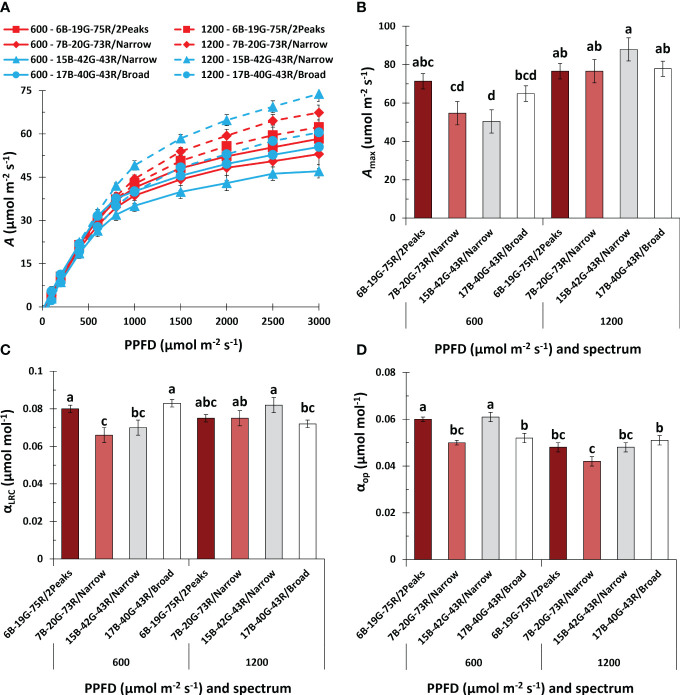
Effects of spectrum and PPFD on leaf net photosynthesis rate (*A*) in the fourth week of the short-day phase of *Cannabis sativa*. **(A)** light response curve of *A*; **(B)** maximum *A* at saturating PPFD (*A*_max_); **(C)** quantum yield of *A* based on the light response curve (α_LRC_); **(D)** quantum yield of operational *A* under the treatment conditions (α_op_). Data was averaged from six plants within each plot, resulting in a single value for each plot. Bars or symbols indicate means of two blocks (n = 2), with the exception of 7B-20G-73R/Narrow and 17B-40G-43R/Broad in panel **(B, C)** which only consisted of one block. Error bars represent standard error of means (SEM). Different letters indicate significant differences between treatments (Fisher’s unprotected LSD test, *P* = 0.10) Conditions within the fluorometer cuvette were set to 27°C, 60% RH, a fan speed of 10000 rpm, a flow rate of 400 µmol s^-1^, 2000 (LRC) and 1000 (OP) ppm [CO_2_], and spectrum of 20B:80R (LRC).

When measuring light-response curves (LRC) of *A* during the fourth week of flowering, it was remarkable that *A* did not saturate, even at the highest PPFD of 3000 μmol m^-2^ s^-1^, in any of the treatments (**Figure 7A**). Also, the increase in *A* at PPFD<1000 μmol m^-2^ s^-1^ was less pronounced in week seven compared to week four of the short-day phase (**Supplementary Figure S4E**). During week four of the short-day phase, *A*_max_ increased with increasing PPFD in plants grown under 7B-20G-73R/Narrow and 15B-42G-43R/Narrow (**Figure 7B**). During week seven of the short-day phase, *A*_max_ decreased compared to week four, with no effect from spectrum or PPFD (**Supplementary Figure S4F**). Photosynthetic quantum yield as derived from the light-response curves (α_LRC_) increased under white light with a dual red peak of 640 and 660 nm compared to white light with a single red peak at 660 nm, and when spectrum broadness increased (**Figure 7C**) at low PPFD. Conversely, at high PPFD, increasing spectrum broadness reduced α_LRC_. During the seventh week of the short-day phase, there was a noticeable decrease in α_LRC_ as PPFD increased, without any effect of spectrum (**Supplementary Figure S5A**).

Dark respiration (R_d_) and the light compensation point (LCP) were not influenced by spectrum, but increased with PPFD (**Supplementary Figure S4A, C**). In the seventh week of the short-day phase, neither spectrum nor PPFD affected R_d_ and LCP (**Supplementary Figure S4B, D**). In week seven of the short-day phase at low PPFD, R_d_,LCP, and *A*_max_ remained relatively stable (**Supplementary Figure S4B, D, F**). However, at high PPFD, these parameters approximately halved compared to week four, suggesting a decline in photosynthetic capacity as leaves aged at high PPFD.

Leaf absorptance within the 400-750 nm range averaged 83% and was unaffected by treatments (**Supplementary Table S4**). The quantum yield of operational photosynthesis under treatment conditions (α_op_) increased at low PPFD under white light with a dual red peak of 640 and 660 nm compared to white light with a single red peak at 660 nm, and when increasing the white fraction (**Figure 7D**). There was no effect of spectrum on α_op_ at high PPFD. In the seventh week of the short-day phase, increasing the PPFD decreased α_op_, with no effect of spectrum (**Supplementary Figure S5B**).

## Discussion

The aim of this study was to investigate the effects of different wavelengths of red (640 and 660 nm), white fraction, and spectrum broadness on the growth and PSM accumulation in *Cannabis sativa*. An in-depth analysis of plant morphology and photosynthetic responses was conducted to elucidate the underlying mechanisms responsible for observed treatment effects.

### White light with dual red peaks at 640 and 660 nm increases inflorescence weight through increased plant dry matter production compared to white light with single red peak At 660 nm

White light with a dual red peak of 640 and 660 nm (6B-19G-75R/2Peaks) increased inflorescence weight (**Figure 4A**) and light use efficiency (LUE; **Figure 4C** and **Supplementary Figure S2B**), compared to white light with a single red peak at 660 nm (7B-20G-73R/Narrow). Similar results were obtained by (Wollaeger and Runkle, 2013) in various ornamental crops. In their study, crops were grown at 125 and 250 µmol m^-2^ s^-1^ PPFD with various combinations of 634 and 664 nm, making up 80% of the spectrum, with 10% blue (446 nm) and 10% green (516 nm). They observed that shoot fresh weight was higher when grown at 40% 634 and 40% 664 nm in comparison to other spectrum combinations, with leaf chlorophyll concentrations being higher under this treatment at low PPFD. Although the light treatment in our study with dual red peak (640 and 660 nm), and single red peak (660 nm) had an equivalent red fraction, the inclusion of two maximum absorption peaks at 640 and 660 nm appeared to drive photosynthesis (α_LRC_ and α_op_) and plant dry matter production more effectively than a single maximum absorption peak at 660 nm (**Figure 7C, D**). This effect may be attributed to the fact that, within the red waveband, Chl *b* and Chl *a* have their maximum absorption peaks around 642 nm and 663 nm, respectively (Zhu et al., 2008; Chazaux et al., 2022). Chl *b* is specifically bound to light-harvesting complexes while Chl *a* is bound to both photosystem core- and light-harvesting complexes (Caffarri et al., 2011; Zhao et al., 2017; Pan et al., 2018). Chl *b* is critical in regulating the size of the light-harvesting complexes, absorbing light energy that would otherwise cause photoinhibition when directly absorbed by the photosystem core complexes (Voitsekhovskaja and Tyutereva, 2015). Distributing the light energy over both Chl *b* and Chl *a* likely allowed for more efficient light energy absorption and conversion to chemical energy, preventing photoinhibition due to excessive light energy. However, Chl *a* and Chl *b* coexist alongside both photosynthetic and non-photosynthetic pigments. This assortment of pigments influences the efficacy of various light wavelengths in driving photosynthesis (Walla et al., 2014; Smith et al., 2017).

The use of 660 nm light may trigger phytochrome activation, inhibiting flowering in short-day plants like strawberries (Takeda and Newell, 2006). This phenomenon could explain the reduced inflorescence weights observed under white light with a single red peak at 660 nm compared to white light with a dual red peak of 640 and 660 nm, likely resulting from a prolonged flower induction phase. Park and Runkle (2018) observed a similar response, where inflorescence buds appeared earlier in begonia (*Begonia* spp.), geranium (*Pelargonium* spp.), petunia (*Petunia* spp.), and snapdragon (*Antirrhinum majus*) under 100% white, or 75% white with 25% red light, compared to a combination of 15% blue and 85% red. Although we did not measure the phytochrome stationary state or the precise moment of flower induction, these factors merit consideration in future research exploring the effects of 640 and 660 nm wavelengths on inflorescence development of medical cannabis.

### Larger fraction of white light improves dry matter partitioning to the inflorescences, but did not increase plant dry matter production

White fraction did not affect inflorescence weight. Increasing the white fraction in our study caused increases in both blue and green fractions and decrease in red fraction. All these changes in fraction blue, green, and red or their mutual ratios could have contributed to the observed treatment effects. Our study, along with similar research in the field, was conducted at low to average PPFD compared to conventional medical cannabis cultivation (Lumigrow, 2017; Fluence, 2020). We found that leaf-level *A* still increased at PPFD >1200 µmol m^-2^ s^-1^, suggesting the potential for further exploration at higher PPFD. While high PPFD can overexcite the photosystems and induce stress responses that give rise to destructive reactive-oxygen-species (ROS) (Demmig-Adams and Adams, 1992; Asada, 2006), increasing the white fraction at high PPFD may alleviate this stress due to a larger green fraction, which penetrates deeper in the leaf and thus distributes light more evenly among the chloroplasts, referred to as the ‘detour’ effect (Terashima et al., 2009; Brodersen and Vogelmann, 2010; Slattery et al., 2016; Smith et al., 2017). In support of this, Liu & Van Iersel (2021) observed higher quantum yields in lettuce under low-white light at 200 µmol m^-2^ s^-1^, and under a combination of red and green light at 1000 µmol m^-2^ s^-1^. Similarly, substituting up to 24% of red+blue LED light with green light increased both shoot fresh and dry weight, which was attributed to green light penetrating deeper within folded lettuce leaves (Kim et al., 2004; Bian et al., 2016).

When PPFD increases, light energy is rarely a limiting factor for plant dry matter production. Nevertheless, overexcitation of pigments can lead to the formation of ROS, potentially causing photooxidative damage to the photosystems and ultimately photoinhibition (Bassi and Dall’Osto, 2021). Up to 90% of red and blue light can be absorbed by the chloroplasts located within the upper 20% of the leaf’s profile (Nishio et al., 1993). Supplementing saturating white halogen light with monochromatic green light enhanced *A* in *Helianthus annuus* more efficiently than monochromatic red light (Terashima et al., 2009). As such, increasing the green fraction is especially relevant in crops which form dense canopies, and in crops that can be grown at very high PPFD, such as medical cannabis (Smith et al., 2017).

Decreasing the white fraction led to an increase in plant height, which is associated with an increased inflorescence weight. Increased plant height results in a more open plant structure. Such open structures have been associated with increased yields and the production of plant specialized metabolites in several plant species (Bugbee, 2016; Danziger and Bernstein, 2021b). The increase in plant height, which led to a more open plant structure, likely increased light distribution in the canopy and photon capture, thereby increasing both whole-crop photosynthesis and plant dry matter production (Takenaka, 1994; Sarlikioti et al., 2011). This factor is particularly vital for medical cannabis, a crop with a dense canopy. A larger white fraction increased dry matter allocation to the inflorescences. This finding is consistent with Magagnini et al. (2018), who reported a lower harvest index with increased red fraction. The effect was ascribed to increased dry matter partitioning to the stems, correlating with an increased plant height. This response aligns with findings by Danziger and Bernstein (2021a), who noted a similar response in plants grown under a high red fraction.

Total cannabinoid concentrations were unaffected by spectrum and PPFD. These observations contradict with those of Islam et al. (2022), who reported increased cannabinoid concentrations under spectra with an increased blue-to-red ratio at a PPFD of 300 µmol m^-2^ s^-1^. Furthermore, in *Mentha* spp., Sabzalian et al. (2014) reported that a combination of red and blue light led to increased essential oil concentrations compared to white light. Studies by Hawley et al. (2018) and Namdar et al. (2018) associated higher blue fractions with increased cannabinoid concentrations. Nevertheless, due to differing experimental conditions, including lower PPFD and shorter durations of the short-day phase, a direct comparison with our findings warrants caution. Westmoreland et al. (2021) and Wei et al. (2021) observed no significant impact of blue fraction on cannabinoid concentrations, and suggested that photoreceptor saturation at high PPFD might underlie these observations. Magagnini et al. (2018) demonstrated that the influence of spectrum on concentrations of THC, CBD, and CBG is cultivar dependent. For instance, Danziger and Bernstein (2021a) observed varying effects on the naturally occurring forms of cannabinoids—Cannabigerolic Acid (CBG), Cannabidiolic Acid (CBD), and Tetrahydrocannabinolic Acid (THC)—across three cultivars when comparing various LED spectra with high-pressure sodium (HPS) lights.

We hypothesize that low-white spectra at high PPFD could overexcite the photosystems, potentially leading to bleached inflorescences, which have been compared to photoinhibition of the leaves, potentially caused by production of reactive oxygen species. A somewhat similar response was observed by (Massa et al., 2008), where white tissue development was observed in peppers grown under 85% red and 15% blue light, specifically on inflorescence sepals. The precise mechanism behind this phenomenon is still uncertain and warrants further investigation. In our research, bleached inflorescences had higher total cannabinoid concentrations, primarily due to more CBD. This could be due to cannabinoids being proposed as potent antioxidants (Mukhopadhyay et al., 2011; Raja et al., 2020), possibly accumulating in greater amounts in tissues with higher concentrations of ROS, to maintain a balance in light-harvesting and energy utilization (Islam et al., 2022).

### No clear effect of spectrum broadness on plant dry matter production and photosynthetic efficiency

Despite a scarcity of studies on the effects of broadband versus narrowband wavelengths, some studies report that plant dry matter production tends to increase under broadband light compared to red and blue light combinations alone (Kim et al., 2004; Massa et al., 2008; Hogewoning et al., 2010; Lu et al., 2019). Spectrum in the PPFD waveband is typically categorized by blue (400-500 nm), green (500-600 nm), and red (600-700 nm) wavelengths. For a comprehensive comparison in peer-reviewed studies, a more detailed classification of broadband wavelengths would be useful, as this could aid in accurately evaluating and contrasting the effects of different spectra on plant dry matter production.

Kim et al. (2004) studied the effects of different spectra, particularly of green fraction, on lettuce growth. They observed that a spectrum with 15% blue, 24% green, and 61% red light led to the highest plant dry matter production compared to cool-white fluorescent light. However, their PPFD of 150 μmol m^-2^ s^-1^ may not have fully demonstrated broadband spectrum potential. Green photons could exhibit a quantum yield similar to red photons, and higher than blue photons, as blue is also absorbed by non-photosynthetic flavonoids and carotenoids (McCree, 1971; Hogewoning et al., 2012). At higher PPFD, a greater green fraction may be more advantageous, as it can enhance light penetration within leaves and through the canopy, which has been hypothesized to improve whole-crop photosynthesis. Johkan et al. (2012) supported this, noting that while low PPFD (100 µmol m^-2^ s^-1^) green light did not significantly impact lettuce growth, higher PPFD (300 µmol m^-2^ s^-1^) green light enhanced growth compared to white fluorescent light. However, contradicting results on the effect of green fraction on plant dry matter production have also been reported (Wang and Folta, 2013; Snowden et al., 2016), which among other factors, could have been attributed to the reversal of blue-light induced stomatal opening (Frechilla et al., 2000; Talbott et al., 2002). The variability in the effects of green fraction on plant dry matter production warrants further investigation. Although green LEDs exhibit inefficiencies in the conversion of electricity to photons—referred to as the ‘green gap’ (Pleasants, 2013)—employing white LEDs or a combination of red and blue LEDs could offer a more effective solution for generating a broad light spectrum.

It is important to note that the broad-white spectrum used in our study included 3% far-red light, while the narrow-white spectrum did not (**Supplementary Table S1**). This is relevant considering that increasing the far-red fraction has been shown lead to an increased growth (Demotes-Mainard et al., 2016). The photosynthetic efficiency of far-red depends on the exact wavelengths used, but can be comparable to PPFD when used in combination with shorter wavelengths (Zhen and Bugbee, 2020a; Zhen and Bugbee, 2020b; Jin et al., 2021). Further research is needed to clarify how the crop responds to different fractions of blue, green, red and far-red light at varying PPFD. Additionally, it is crucial to investigate whether the crop’s light requirements change during different stages of development.

## Conclusions

Our study revealed an interaction between spectrum and PPFD on plant dry matter production and inflorescence yield of medical cannabis. White light with a dual red peak at 640 and 660 nm, compared to white light with a single red peak at 660 nm, increased inflorescence yield and light use efficiency, regardless of PPFD. This increase was primarily due to increased total plant dry matter production and a more open plant architecture, which may have improved photon capture. White fraction and spectrum broadness had no effect on inflorescence yield, irrespective of PPFD. There was no treatment effect on total cannabinoid concentrations, which indicates a promising potential for maintaining consistent quality in terms of PSM. However, at higher PPFD, white light with a dual red peak of 640 and 660 nm compared to white light with a single red peak at 660 nm increased terpenoid concentrations. At low PPFD, photosynthetic parameters like maximum photosynthetic rate and quantum yield were increased when grown under white light with a dual red peak of 640 and 660 nm compared to white light with a single red peak at 660 nm, while spectrum had no effect at higher PPFD. The addition of 640 nm alongside 660 nm shows potential in improving light use efficiency and promoting plant dry matter production.

## Data availability statement

The original contributions presented in the study are included in the article/**Supplementary Material**. Further inquiries can be directed to the corresponding authors.

## Author contributions

MH: Conceptualization, Data curation, Formal analysis, Investigation, Methodology, Visualization, Writing – original draft, Writing – review & editing, Software, Validation. EK: Methodology, Writing – review & editing, Supervision. IK: Methodology, Writing – review & editing, Supervision. EH: Supervision, Writing – review & editing, Methodology. LM: Conceptualization, Funding acquisition, Supervision, Writing – review & editing, Methodology.
