# Supplementary material for: The role of red and white light in optimizing growth and accumulation of plant specialized metabolites at two light intensities in medical cannabis (Cannabis sativa L.)
Source: Front Plant Sci. 2024 Jun 18;15:1393803. doi: 10.3389/fpls.2024.1393803 (PMC11217568; doi:10.3389/fpls.2024.1393803)
Supplement: Supplementary file 1 [file DataSheet_1.docx]

# Supplementary

**Supplementary Text 1 |** Propagation protocol

Mother plants were cultivated at a PPFD of 300 µmol m^-2^ s^-1^ (SPYDR LED; Fluence, Texas, Austin, USA, with 20% blue (B, 400-500nm), 40% green (G, 500-600nm) and 40% red (R, 600-700nm)). Growth conditions were set to 16/8 h (day/night) photoperiod, 24/18 °C (day/night) air temperature, 65% relative humidity (RH), and no CO_2_ was supplied. Unrooted cuttings were stored in darkness at 6 °C for 24 hours in sealed plastic containers to promote root formation and to inhibit desiccation. Cuttings were then dipped in rooting powder (IBA 0.25% w/w%; Rhizopon Group, Hazerswoude-Rijndijk, the Netherlands) and inserted into 3.6 x 3.6 x 4 cm stone wool plugs (VAO plug; Grodan, Roermond, the Netherlands). These were placed in 78-cell nursery trays (Gro-Smart Trays; Grodan) at a planting density of 252 plants m^-2^, and covered with plastic domes. Subsequently, cuttings were positioned 20 cm beneath LED fixtures (GreenPower LED Low Blue. Generation 2.; 13B:17G:70R; Philips, Eindhoven, the Netherlands) and subjected to a 18/6 h photoperiod, while air temperature was set to 22/22 °C, relative air humidity to 95%, and [CO_2_] to 450 ppm. PPFD at canopy height was 80 µmol m^-2^ s^-1^. Upon the emergence of root tips – typically observed 10-15 days post-insertion – plastic domes were removed, PPFD at canopy height was increased to 220 µmol m^-2^ s^-1^ and RH decreased to 80% to promote plant dry matter production and transpiration.

**Supplementary Table 1 |** Nutritional regimen of Cannabis sativa across different growth phases: propagation, long-day, and short-day. Electrical conductivity (EC) in dS m^-1^, pH, macronutrient (NH_4_, NO_3_, P, K, Ca, Mg, SO_4_) and micronutrient concentrations (B, Fe, Mn, Cu, Zn, Mo) in mmol L^-1^ or µmol L^-1^, are shown.

| **crop phase** | **propagation** | **long-day phase** | **short-day phase** |
| --- | --- | --- | --- |
|  |  |  |  |
| EC | 1.5 | 2.2 | 2.5 |
| pH | 5.5 | 5.5 | 5.5 |
|  | **mmol L^-1^** | **mmol L^-1^** | **mmol L^-1^** |
| **NH_4_** | 1.25 | 1.8 | 2 |
| **NO_3_** | 10.5 | 13.3 | 15.1 |
| **P** | 0.8 | 2 | 2.3 |
| **K** | 6.2 | 6 | 6.8 |
| **Ca** | 1.9 | 3.8 | 4.3 |
| **Mg** | 0.9 | 3.2 | 3.6 |
| **SO_4_** | 0.8 |  |  |
|  | **µmol L^-1^** | **µmol L^-1^** | **µmol L^-1^** |
| **B** | 20 | 40 | 40 |
| **Fe** | 60 | 60 | 60 |
| **Mn** | 20 | 10 | 10 |
| **Cu** | 0.5 | 1 | 1 |
| **Zn** | 3 | 3 | 3 |
| **Mo** | 0.5 | 0.5 | 0.5 |

**Supplementary Table 2 |** Effects of spectrum and PPFD on the concentration of specific cannabinoids (THC, CBD, CBN, CBG, CBC, Δ8-THC) and terpenoids (β-Myrcene, Limonene, α-Pinene, β-Pinene, β-Caryophyllene, α-Humulene, α-Bergamotene, Germacrene D, 4-Epicubebol, α-Eudesmol, Selinene, Monoterpenoids, Sesquiterpenoids, Total Terpenoids) of *Cannabis* *sativa*. Data were analyzed for significant differences due to light treatment, spectrum, and their interaction, with *P* values reported. Values indicate means of two blocks (n = 2) each consisting of 9 replicate plants. Error bars represent standard error of means (SEM). Different letters indicate significant differences between treatments (Fisher’s unprotected LSD test, *P* = 0.10)

|  | **600** | **1200** | **600** **µmol m^-2^ s^-1^** | | | | **1200** **µmol m^-2^ s^-1^** | | | | ***P* value** | | |
| --- | --- | --- | --- | --- | --- | --- | --- | --- | --- | --- | --- | --- | --- |
|  |  |  | **6B-19G-75R/2Peaks** | **7B-20G-73R/Narrow** | **15B-42G-43R/Narrow** | **17B-40G-43R/Broad** | **6B-19G-75R/2Peaks** | **7B-20G-73R/Narrow** | **15B-42G-43R/Narrow** | **17B-40G-43R/Broad** | **Light** | **Spectrum** | **Light * Spectrum** |
| **THC**  **(mg g^-1^)** | 73  ±6 | 76  ±6 | 84  ±12 | 80  ±12 | 63  ±12 | 64  ±12 | 74  ±12 | 69  ±12 | 75  ±12 | 84  ±12 | 0.756 | 0.875 | 0.551 |
| **CBD**  **(mg g^-1^)** | 61  ±6 | 62  ±6 | 74  ±12 | 67  ±12 | 51  ±12 | 56  ±12 | 60  ±12 | 56  ±12 | 60  ±12 | 74  ±12 | 0.839 | 0.816 | 0.480 |
| **CBN**  **(mg g^-1^)** |  |  | 0.5 |  | 0.2 |  |  | 0.2 | 0.2 | 0.4 |  |  |  |
| **CBG**  **(mg g^-1^)** | 0.4  ±0.1 | 0.7 ±0.1 | 0.4  ±0.1 | 0.3  ±0.1 | 0.01  ±0.2 | 1.0  ±0.2 | 0.2**B**  ±0.1 | 0.5**AB**  ±0.1 | 0.4**AB**  ±0.1 | 1.5**A**  ±0.1 | 0.323 | 0.186 | 0.482 |
| **CBC**  **(mg g^-1^)** | 3.5  ±0.5 | 3.8 ±0.5 | 3.9  ±1 | 4.2  ±1 | 2.7  ±1 | 3.1  ±1 | 3.7  ±1 | 4.0  ±1 | 3.3  ±1 | 4.5 ±1 | 0.643 | 0.740 | 0.816 |
| **Δ8-THC**  **(mg g^-1^)** | 0.4  ±0.1 | 0.4 ±0.1 | 0.5  ±0.1 | 0.4  ±0.1 | 0.2  ±0.1 | 0.4  ±0.2 | 0.3  ±0.1 | 0.3  ±0.1 | 0.3  ±0.1 | 0.5  ±0.1 | 0.749 | 0.511 | 0.669 |
| **Total Cannabinoids**  **(mg g^-1^)** | 137.4 ±13 | 143.1 ±13 | 164  ±26 | 151  ±26 | 116  ±26 | 117  ±26 | 138  ±26 | 129  ±26 | 139  ±26 | 164  ±26 | 0.775 | 0.846 | 0.519 |
| **β-Myrcene**  **(mg g^-1^)** | 3.3  ±0.5 | 2.8 ±0.5 | 3  ±1.1 | 3.3  ±1.1 | 3.5  ±1.1 | 3.6  ±1.1 | 5.5**A**  ±1.1 | 1.6**B**  ±1.1 | 2.6**AB**  ±1.1 | 1.4**B**  ±1.1 | 0.497 | 0.425 | 0.295 |
| **Limonene**  **(mg g^-1^)** | 0.4 ±0.04 | 0.4 ±0.04 | 0.4  ±0.09 | 0.4  ±0.09 | 0.4  ±0.09 | 0.4  ±0.09 | 0.6**A**  ±0.09 | 0.2**B**  ±0.09 | 0.3**B**  ±0.09 | 0.2**B**  ±0.09 | 0.636 | 0.275 | 0.230 |
| **α-Pinene**  **(mg g^-1^)** | 1.8  ±0.3 | 1.6 ±0.3 | 1.3  ±0.6 | 2  ±0.6 | 1.9  ±0.6 | 1.8  ±0.6 | 3.1**A**  ±0.6 | 1.0**B**  ±0. | 1.6**AB**  ±0.6 | 0.8**B**  ±0.6 | 0.780 | 0.536 | 0.232 |
| **β-Pinene**  **(mg g^-1^)** | 0.8  ±0.1 | 0.7 ±0.1 | 0.6  ±0.3 | 0.8  ±0.3 | 0.8  ±0.3 | 0.8  ±0.3 | 1.3**A**  ±0.3 | 0.4**B**  ±0.3 | 0.7**AB**  ±0.3 | 0.3**B**  ±0.3 | 0.706 | 0.468 | 0.276 |
| **β-Caryophyllene**  **(mg g^-1^)** | 0.5  ±0.1 | 0.5 ±0.1 | 0.4  ±0.1 | 0.5  ±0.1 | 0.5  ±0.1 | 0.5  ±0.1 | 0.7  ±0.1 | 0.4  ±0.1 | 0.4  ±0.1 | 0.4  ±0.1 | 0.875 | 0.629 | 0.425 |
| **α-Humulene**  **(mg g^-1^)** | 0.3 ±0.03 | 0.2 ±0.03 | 0.3  ±0.06 | 0.3  ±0.06 | 0.2  ±0.06 | 0.3  ±0.06 | 0.3  ±0.06 | 0.2  ±0.03 | 0.2  ±0.03 | 0.2  ±0.03 | 0.528 | 0.496 | 0.594 |
| **α-Bergamotene**  **(mg g^-1^)** | 0.2 | 0.1 | 0.2 | 0.2 |  | 0.2 | 0.2 | 0.1 | 0.2 | 0.1 |  |  |  |
| **Germacrene D**  **(mg g^-1^)** | 0.5 ±0.04 | 0.4 ±0.04 | 0.4  ±0.1 | 0.5  ±0.1 | 0.4  ±0.1 | 0.6  ±0.1 | 0.7**A**  ±0.1 | 0.3**B**  ±0.1 | 0.4**AB**  ±0.1 | 0.3**B**  ±0.1 | 0.480 | 0.498 | 0.194 |
| **4-e*pi-C*ubebol**  **(mg g^-1^)** | 0.2 ±0.02 | 0.2 ±0.02 | 0.2  ±0.03 | 0.2  ±0.03 | 0.2  ±0.03 | 0.2  ±0.03 | 0.2  ±0.03 | 0.2  ±0.03 | 0.2  ±0.07 | 0.2  ±0.03 | 0.632 | 0.900 | 0.617 |
| **α-Eudesmol**  **(mg g^-1^)** | 0.2 ±0.02 | 0.2 ±0.01 | 0.2  ±0.02 | 0.2  ±0.03 | 0.2  ±0.04 | 0.2  ±0.02 | 0.2  ±0.02 |  | 0.2  ±0.02 | 0.2  ±0.02 | 0.667 | 0.485 | 0.544 |
| **Selinene**  **(mg g^-1^)** | 0.3 ±0.03 | 0.3 ±0.03 | 0.3  ±0.1 | 0.3  ±0.1 | 0.2  ±0.1 | 0.2  ±0.1 | 0.3  ±0.1 | 0.3  ±0.1 | 0.2  ±0.1 | 0.3  ±0.1 | 0.901 | 0.521 | 0.758 |
| **Monoterpenoids**  **(mg g^-1^)** | 6.2  ±0.9 | 5.4 ±0.9 | 5.3  ±1.8 | 6.6  ±1.8 | 6.5  ±1.8 | 6.5  ±1.8 | 10.6**A**  ±1.8 | 3.2**B**  ±1.8 | 5.3**AB**  ±1.8 | 2.7**B**  ±1.8 | 0.575 | 0.402 | 0.227 |
| **Sesquiterpenoids**  **(mg g^-1^)** | 1.9  ±0.2 | 1.8 ±0.2 | 1.8  ±0.4 | 2.0  ±0.4 | 1.6  ±0.4 | 2.0  ±0.4 | 2.6  ±0.4 | 1.4  ±0.4 | 1.6  ±0.4 | 1.6  ±0.4 | 0.865 | 0.600 | 0.470 |
| **Total Terpenoids**  **(mg g^-1^)** | 8.1  ±1.1 | 7.2 ±1.1 | 7.1  ±2.2 | 8.1  ±2.2 | 8.6  ±2.2 | 8.6  ±2.2 | 13.1**A**  ±2.2 | 6.9**B**  ±2.2 | 4.6**AB**  ±2.2 | 4.2**B**  ±2.2 | 0.622 | 0.448 | 0.258 |

**Supplementary Table 3 |** Effects of flower type on the concentration of cannabinoids (THC, CBD, CBG, CBC, Δ8-THC, Total Cannabinoids) and terpenoids (β-Myrcene, Limonene, α-Pinene, β-Pinene, β-Caryophyllene, α-Humulene, α-Bergamotene, Germacrene D, 4-*epi*-Cubebol, α-Eudesmol, Selinene, Monoterpenoids, Sesquiterpenoids, Total Terpenoids) of *Cannabis sativa*. Data were analyzed for significant differences due to light treatment, spectrum, and their interaction, with P values reported. Values indicate means of one block (n = 1) each consisting of 9 replicate plants. Error bars represent standard error of means (SEM).

|  | **Green Flower** | **Bleached Flower** | ***P* value** |
| --- | --- | --- | --- |
| **THC**  **(% mg g^-1^)** | 57  ±7 | 109  ±27 | 0.161 |
| **CBD**  **(% mg g^-1^)** | 46  ±8 | 132  ±29 | **0.063** |
| **CBG**  **(% mg g^-1^)** |  |  |  |
| **CBC**  **(% mg g^-1^)** | 0.8  ±0.2 | 4.1  ±0.5 | **0.080** |
| **Δ8-THC**  **(% mg g^-1^)** | 2.9  ±0.5 | 6.3  ±1.7 | 0.150 |
| **Total**  **Cannabinoids**  **(% mg g^-1^)** | 106  ±17 | 251  ±58 | 0.099 |
| **β-Myrcene**  **(% mg g^-1^)** | 3.5  ±0.5 | 2.2  ±1.9 | 0.531 |
| **Limonene**  **(% mg g^-1^)** | 0.4  ±0.1 | 0.3  ±0.2 | 0.535 |
| **α-Pinene**  **(% mg g^-1^)** | 2.3  ±0.5 | 2.9  ±1.7 | 0.756 |
| **β-Pinene**  **(% mg g^-1^)** | 1  ±0.2 | 1  ±0.7 | 0.770 |
| **β-Caryophyllene**  **(% mg g^-1^)** | 0.5  ±0.1 | 0.9  ±0.2 | 0.138 |
| **α-Humulene**  **(% mg g^-1^)** | 0.2  ±0.04 | 0.5  ±0.12 | 0.174 |
| **α-Bergamotene**  **(% mg g^-1^)** |  |  |  |
| **Germacrene D**  **(% mg g^-1^)** | 0.4  ±0.1 | 1  ±2 | 0.110 |
| **4-*epi*-Cubebol**  **(% mg g^-1^)** | 0.2  ±0.02 | 0.3  ±0.06 | 0.126 |
| **α-Eudesmol**  **(% mg g^-1^)** | 0.2  ±0.02 | 0.3  ±0.07 | 0.329 |
| **Selinene**  **(% mg g^-1^)** | 0.2  ±0.03 | 0.04  ±0.010 | 0.146 |
| **Monoterpenoids**  **(% mg g^-1^)** | 0.72  ±0.13 | 0.66  ±0.44 | 0.890 |
| **Sesquiterpenoids**  **(% mg g^-1^)** | 0.17  ±0.03 | 0.34  ±0.09 | 0.150 |
| **Total Terpenes**  **(% mg g^-1^)** | 0.9  ±0.2 | 1.0  ±0.5 | 0.862 |

**Supplementary Table 4 |** Leaf light transmittance, reflectance, and absorptance (as fractions of 1) of *Cannabis sativa* under the studied light treatments (6B-19G-75R/2Peaks (1), 7B-20G-73R/Narrow (2), 15B-42G-43R/Narrow (3), 17B-40G-43R/Broad (4)) at two PPFD levels (600 and 1200 µmol m^-2^ s^-1^).

|  | **600** **µmol m^-2^ s^-1^** | | | | **1200** **µmol m^-2^ s^-1^** | | | |
| --- | --- | --- | --- | --- | --- | --- | --- | --- |
| **Spectra** | **1** | **2** | **3** | **4** | **1** | **2** | **3** | **4** |
| **Transmittance** | 0.05 | 0.05 | 0.05 | 0.04 | 0.05 | 0.06 | 0.05 | 0.06 |
| **Reflectance** | 0.11 | 0.11 | 0.11 | 0.11 | 0.11 | 0.12 | 0.12 | 0.12 |
| **Absorptance** | 0.84 | 0.83 | 0.85 | 0.85 | 0.84 | 0.82 | 0.83 | 0.82 |

##

**Supplementary Figure 1 |** Effects of spectrum on plant morphology at intermediate harvest (switch from long-day to short-day phase) of *Cannabis sativa*. A, plant height; B, specific leaf area; C, leaf area; D, leaf area index; and E, plant dry weight and partitioning. Bars indicate means of two blocks (n = 2) each consisting of 7 replicate plants. Error bars represent standard error of means (SEM). Different letters indicate significant differences between treatments (Fisher’s unprotected LSD test, *P* = 0.10)

**Supplementary Figure 2 |** Effects of spectrum and PPFD on yield and light use efficiency of *Cannabis sativa* at final harvest. A, inflorescence weight at 10% moisture content; B, plant light use efficiency; C, leaf area; D, specific leaf area. Bars indicate means of two blocks (n = 2) each consisting of 9 replicate plants. Main effects are shown when no interaction is found. Error bars represent standard error of means (SEM). Different letters (within lowercase and uppercase) indicate significant differences between treatments (Fisher’s unprotected LSD test, *P* = 0.10)

**Supplementary Figure 3 |** Effects of spectrum and PPFD on PSM concentrations and accumulation of *Cannabis* *sativa*. A, total cannabinoid concentration; B, total terpenoid concentration; C, THC yield; D, CBD yield. Bars indicate means of two blocks (n = 2) each consisting of 9 replicate plants, with the exception of 15, 10, 5, and 0 DBH in panel A and B which only consisted of one block. Main effects are shown when no interaction is found. Error bars represent standard error of means (SEM). Different letters (within lowercase and uppercase) indicate significant differences between treatments (Fisher’s unprotected LSD test, *P* = 0.10)

**Supplementary Figure 4 |** Effects of spectrum and PPFD on leaf net photosynthesis rate (*A*) in the fourth (F4) and seventh (F7) week of the short-day phase of *Cannabis* *sativa* and leaf photosynthesis parameters derived from light response curves. A and B, dark respiration (R_d_); C and D, light compensation point (LCP); E, light response curve of *A*; F, maximum *A* at saturating PPFD (*A*_max_)*.* Data was averaged from six plants within each plot, resulting in a single value for each plot. Bars or symbols indicate means of two blocks (n = 2), with the exception of W_low-narrow_R_660_ and W_high-broad_R_660_ in A, C, and all treatments in B, D, E, F which only consisted of one block. Main effects are shown when no interaction is found. Error bars represent standard error of means (SEM). Different letters (within lowercase and uppercase) indicate significant differences between treatments (Fisher’s unprotected LSD test, *P* = 0.10). Conditions within the fluorometer cuvette were set to 27 °C, 60% RH, a fan speed of 10000 rpm, a flow rate of 400 µmol s^-1^, 2000 (LRC) and 1000 (OP) ppm [CO_2_], and spectrum of 20B:80R (LRC).

**Supplementary Figure 5 |** Effects of spectrum and PPFD on leaf net photosynthesis rate (*A*) in the seventh (F7) week of the short-day phase of *Cannabis* *sativa* and leaf photosynthesis parameters derived from light response curves. A, quantum yield of *A* based on the light response curve (α_LRC_); B, quantum yield of operational *A* under the treatment conditions (α_op_). Data was averaged from six plants within each plot, resulting in a single value for each plot. Bars or symbols indicate means of one block (n = 1). Main effects are shown when no interaction is found. Error bars represent standard error of means (SEM). Different letters (within lowercase and uppercase) indicate significant differences between treatments (Fisher’s unprotected LSD test, *P* = 0.10). Conditions within the fluorometer cuvette were set to 27 °C, 60% RH, a fan speed of 10000 rpm, a flow rate of 400 µmol s^-1^, 2000 (LRC) and 1000 (OP) ppm [CO_2_], and spectrum of 20B:80R (LRC).
