# Supplementary figures and images for: The role of red and white light in optimizing growth and accumulation of plant specialized metabolites at two light intensities in medical cannabis (Cannabis sativa L.)
Source: Front Plant Sci. 2024 Jun 18;15:1393803. doi: 10.3389/fpls.2024.1393803 (PMC11217568; doi:10.3389/fpls.2024.1393803)

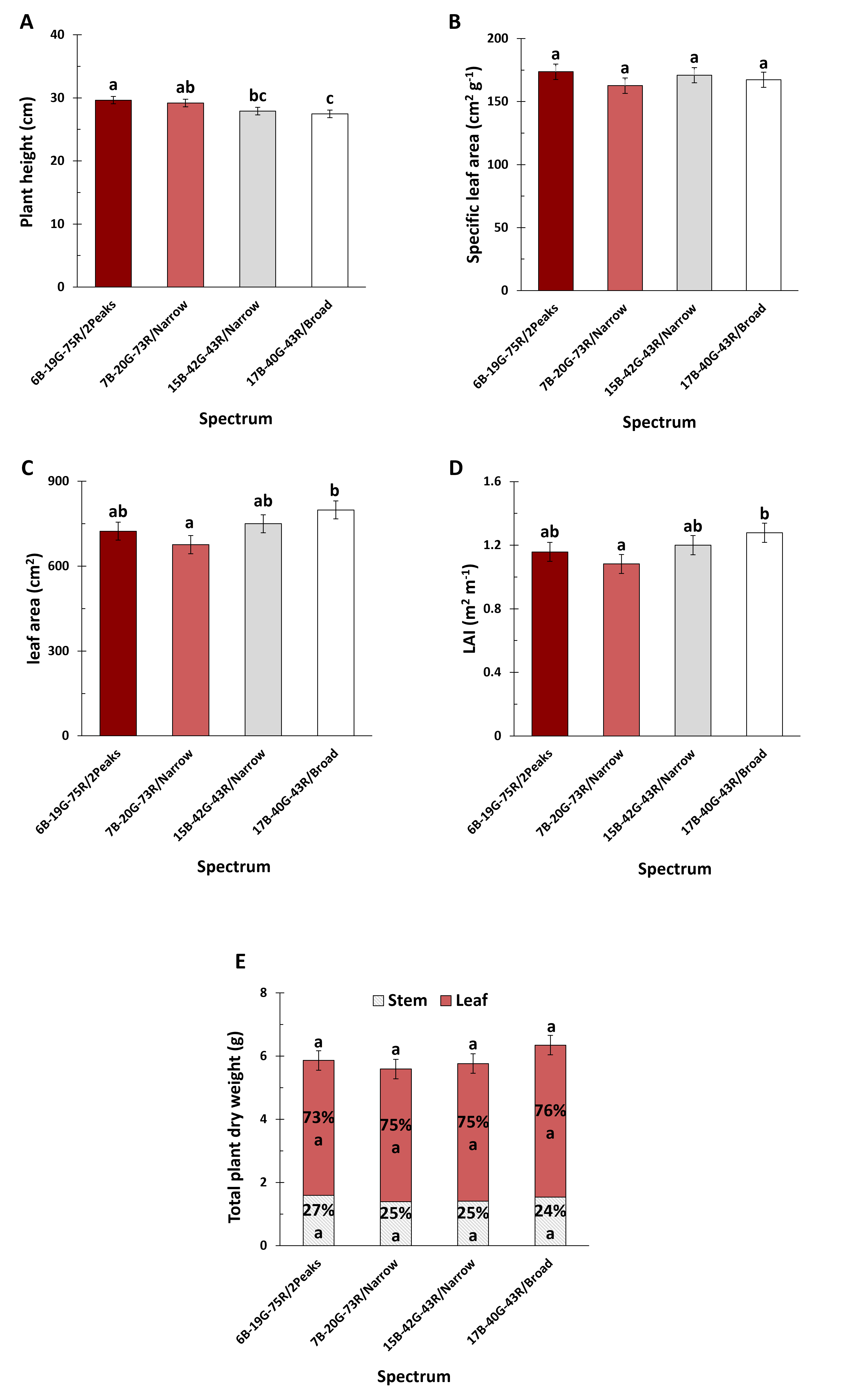

Supplement: Supplementary file 2 [file Image_1.tif]

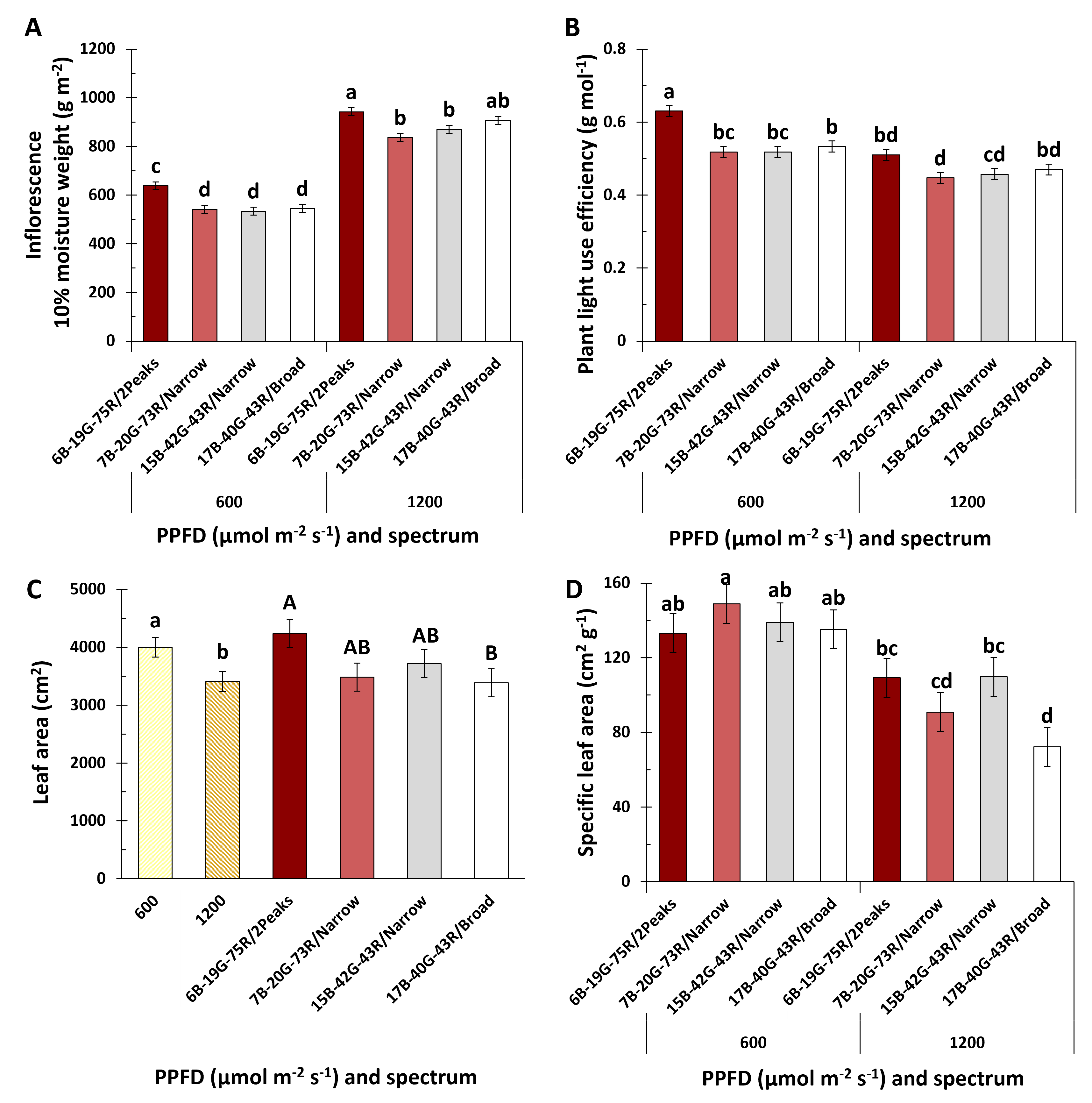

Supplement: Supplementary file 3 [file Image_2.tif]

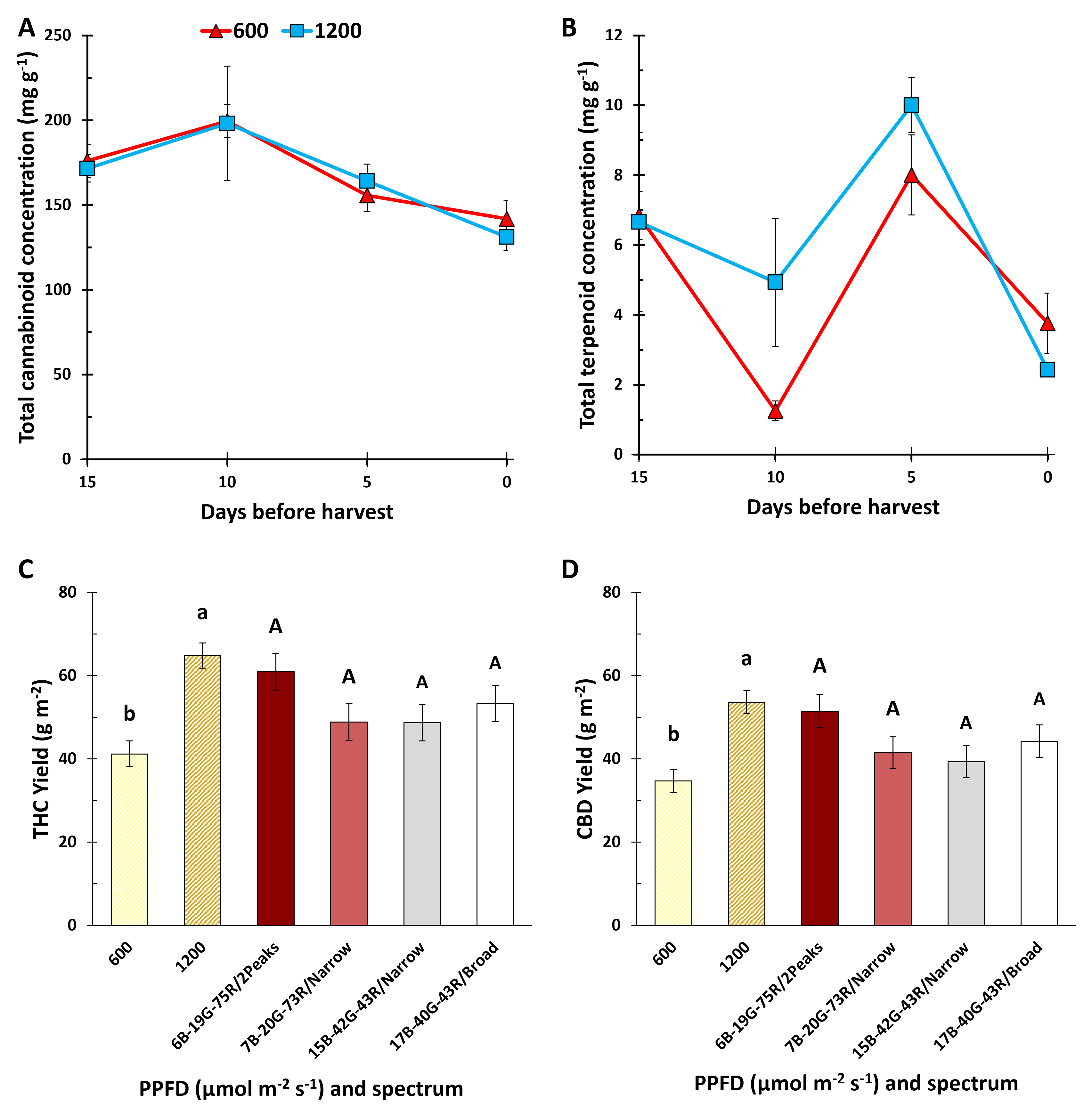

Supplement: Supplementary file 4 [file Image_3.tif]

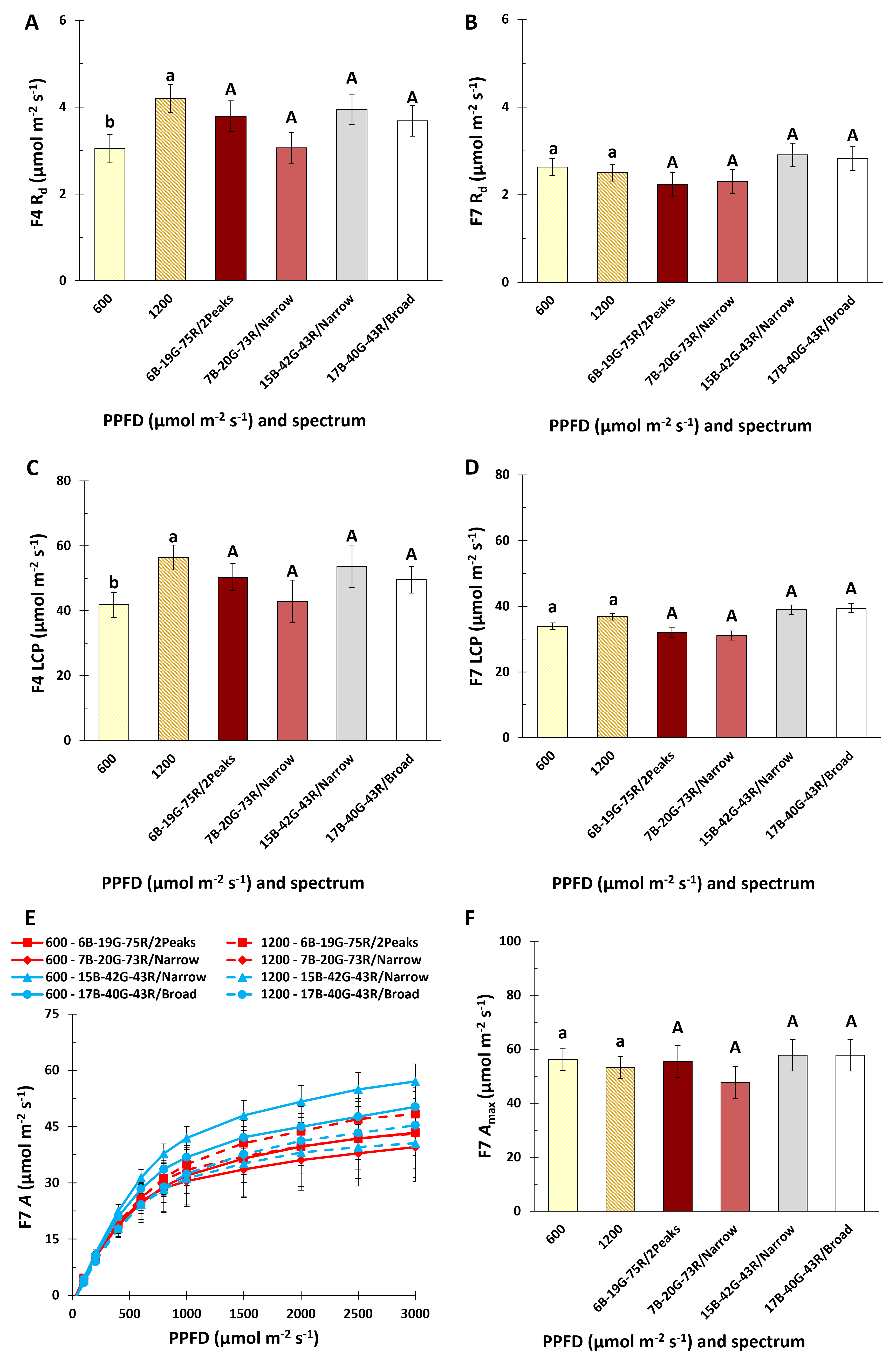

Supplement: Supplementary file 5 [file Image_4.tif]

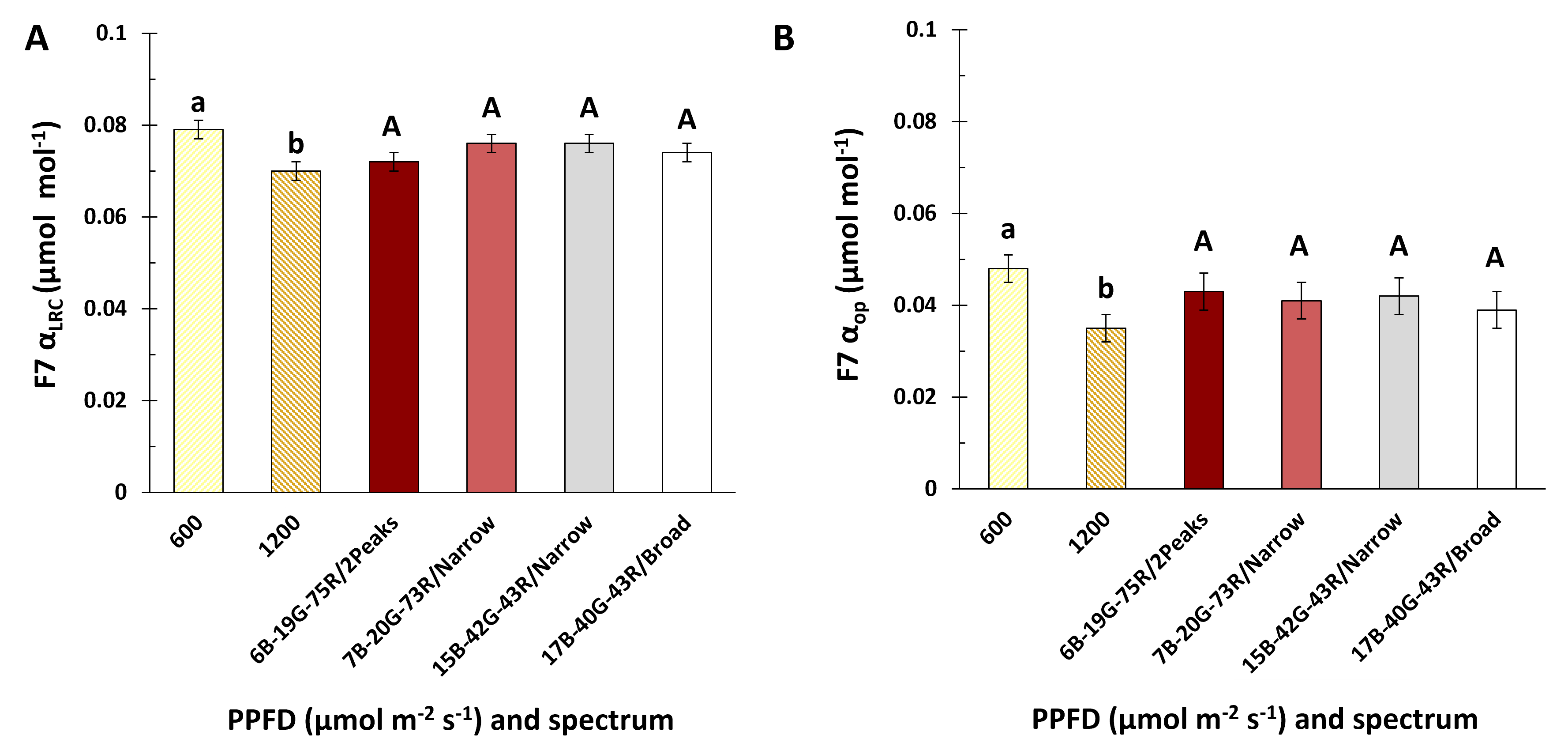

Supplement: Supplementary file 6 [file Image_5.tif]
